# A novel cuproptosis-related LncRNA signature: Prognostic and therapeutic value for acute myeloid leukemia

**DOI:** 10.3389/fonc.2022.966920

**Published:** 2022-10-07

**Authors:** Pian Li, Junjun Li, Feng Wen, Yixiong Cao, Zeyu Luo, Juan Zuo, Fei Wu, Zhiqin Li, Wenlu Li, Fujue Wang

**Affiliations:** ^1^ The First Affiliated Hospital, Department of Oncology Radiotherapy, Hengyang Medical School, University of South China, Hengyang, China; ^2^ The First Affiliated Hospital, Department of Hematology, Hengyang Medical School, University of South China, Hengyang, China; ^3^ Department of Hematology, West China Hospital of Sichuan University, Chengdu, China

**Keywords:** cuproptosis-related Genes (CRGs), long non-coding RNAs (lncRNAs), acute myeloid leukemia (AML), prognostic value, chemotherapy and immunotherapy

## Abstract

**Background:**

Cuproptosis is a type of programmed cell death that is involved in multiple physiological and pathological processes, including cancer. We constructed a prognostic cuproptosis-related long non-coding RNA (lncRNA) signature for acute myeloid leukemia (AML).

**Methods:**

RNA-seq and clinical data for AML patients were acquired from The Cancer Genome Atlas (TCGA) database. The cuproptosis-related prognostic lncRNAs were identified by co-expression and univariate Cox regression analysis. The least absolute shrinkage and selection operator (LASSO) was performed to construct a cuproptosis-related lncRNA signature, after which the AML patients were classified into two risk groups based on the risk model. Kaplan-Meier, ROC, univariate and multivariate Cox regression, nomogram, and calibration curves analyses were used to evaluate the prognostic value of the model. Then, expression levels of the lncRNAs in the signature were investigated in AML samples by quantitative polymerase chain reaction (qPCR). KEGG functional analysis, single-sample GSEA (ssGSEA), and the ESTIMATE algorithm were used to analyze the mechanisms and immune status between the different risk groups. The sensitivities for potential therapeutic drugs for AML were also investigated.

**Results:**

Five hundred and three lncRNAs related to 19 CRGs in AML samples from the TCGA database were obtained, and 21 differentially expressed lncRNAs were identified based on the 2-year overall survival (OS) outcomes of AML patients. A 4-cuproptosis-related lncRNA signature for survival was constructed by LASSO Cox regression. High-risk AML patients exhibited worse outcomes. Univariate and multivariate Cox regression analyses demonstrated the independent prognostic value of the model. ROC, nomogram, and calibration curves analyses revealed the predictive power of the signature. KEGG pathway and ssGSEA analyses showed that the high-risk group had higher immune activities. Lastly, AML patients from different risk groups showed differential responses to various agents.

**Conclusion:**

A cuproptosis-related lncRNA signature was established to predict the prognosis and inform on potential therapeutic strategies for AML patients.

## Introduction

Acute myeloid leukemia (AML) is a highly heterogeneous leukemia that is associated with abnormalities in genetics, epigenetics, and cytogenetics (1). Over the last four decades, chemotherapy has remained the main treatment option for AML, However, most patients exhibit dismal outcomes and less than one-third of adult patients acquired durable remission (2). With the recent advances in molecular biology as well as the discovery of drivers for leukemogenesis and due to a better understanding of the AML, including the tumor environment (TME) and immune landscape, clinical trials, as well as novel therapies are now being promoted (2–4). Therefore, identification of novel prognostic and therapeutic targets will inform on development of personalized therapies for AML patients.

Cuproptosis is a recently defined type of cell death that differs from the well-known programmed cell death types, such as apoptosis, ferroptosis, pyroptosis, and necroptosis (5–8). Cuproptosis is involved in various physiological and pathological processes, including multiple cancers, and both copper ion carriers as well as copper chelators have potent anticancer activity (7). Disulfiram with copper selectively eradicated AML stem cells by activating the ROS-JNK while inhibiting the NF-κB and Nrf2 pathways (9). However, the role cuproptosis and its regulation in AML remains unclear.

Long non-coding RNAs (lncRNAs) are transcripts longer than 200 nucleotides, which are classified as non-coding RNAs, and are involved in the epigenetic regulation of gene expressions (10). They are associated with distinct cell death types and are implicated in several cancer types, including AML (10–15). Various lncRNAs are involved in leukemia and are potential diagnostic or prognostic biomarkers as well as therapeutic targets (16). However, the relationship between cuproptosis-related genes (CRGs) and lncRNAs in leukemia has yet to be reported.

We established a cuproptosis-related lncRNA signature using The Cancer Genome Atlas (TCGA) database to serve as a prognostic marker and to elucidate on the mechanism of cuproptosis in AML patients.

## Materials and methods

### Data collection and processing

RNA-seq data corresponding to the clinical data for 151 AML patients from the TCGA-LAML database were downloaded in the FPKM format (https://portal.gdc.cancer.gov) (17). The annotation of lncRNAs was obtained from the GENCODE (https://www.gencodegenes.org/) (18). Patients diagnosed with the M3 subtype according to the French-American-British (FAB) classification and those without complete clinical information were excluded from this study. Finally, 129 AML patients were included in this study, and their clinicopathological characteristics are shown in **Table S1**. To construct a prognostic cuproptosis-related lncRNA signature, 129 AML samples were randomized into a training set (93 cases) and validation set (36 cases) using “caret” R package.

### Screening for cuproptosis-related lncRNAs

Nineteen CRGs (**Table S2**) were obtained from the literatures (5, 9, 19). Genetic alterations of CRGs in AML patients from the TCGA database were investigated using the cBioPortal database (https://www.cbioportal.org/). Correlations between cuproptosis-related lncRNAs in AML patients were determined using “limma” R package with a correlation coefficient of > 0.4 and *p* < 0.001 were set as the threshold. AML patients in the training set were assigned into two groups according to 2-year overall survival (OS) time based on clinical experience and previous studies (20, 21). Differentially expressed lncRNAs were identified using “DEGseq” R package with *p* < 0.05 and |log fold change (log FC) | ≥ 1 as the filter criteria.

### Construction of a prognostic cuproptosis-related lncRNA signature for AML patients

Univariate Cox regression analysis was performed to screen for cuproptosis-related lncRNAs associated with survival from the identified differentially expressed lncRNAs (FDR < 0.05). Then, LASSO Cox regression analysis was performed with 10-fold cross-validation to establish the cuproptosis-related lncRNA signature. Analysis of the proportional hazards (PH) hypothesis and multicollinearity of covariates estimated by the variance inflation factor (VIF) were performed. LncRNAs that satisfied the PH hypothesis (*p* > 0.05) and VIF < 2 were selected to construct the signature *via* multivariate Cox regression (22). The risk score was calculated as follows:

Risk Score


expression $$=\sum_{i=1}^{n}\beta \times \text{lncRNA}$$

Whereby *β* is the regression coefficient. Regulatory networks of CRGs and the screened lncRNAs were analyzed and displayed using the “ggalluvial” R package.

### Independent prognostic role of the cuproptosis-related lncRNA signature for AML patients

Univariate and multivariate Cox regression analyses were performed to determine the independent prognostic value of the constructed cuproptosis-related lncRNA risk model. Multiple clinical factors, including sex, age, white blood cell counts (WBCs), FAB classification, blasts in bone marrow (BM), and molecular risk stratification, were considered during the analysis. A nomogram for 1-, 3-, and 5-year OS was plotted with the above factors using the “rms” R package, and calibration curves analyses were performed to assess the accuracy of prediction.

### Quantitative polymerase chain reaction (qPCR)

We had previously collected bone marrow samples for 50 newly diagnosed AML patients and peripheral blood samples for 6 healthy volunteers, and conducted a study for *TRPM4* gene in *MLL*-rearranged AML. Informed consents were obtained from all the participants and the Ethical Committee of the West China Hospital of Sichuan University had approved the study (1).

We used the partially reserved cDNA of mononuclear cells from bone marrow or peripheral blood samples to investigate the expressions of lncRNAs in the signature. The qPCR assay was performed using 2 × SYBR Green qPCR Master Mix (Bimake, China, B21203) on a LightCycler 480 II instrument (Roche, Switzerland). Relative expressions of the lncRNAs were normalized to *GAPDH* and calculated using the 2^-ΔΔCt^ method. The primers used in this study are shown in **Table S3**.

### Gene set enrichment analysis (GSEA)

Functional enrichments analysis between the low- and high-risk subgroups were analyzed using the GSEA 4.2.2 software with the curated gene set (kegg.v7.5.symbols.gmt) based on the criterion: |NES| > 1.5, NOM *p* < 0.05 and FDR< 0.25.

### Evaluation of immune infiltration status and tumor environment (TME)

To investigate the immune infiltration status of AML patients with different risk groups, single-sample gene set enrichment analysis (ssGSEA) was performed using “GSVA” R package to calculate the infiltration scores for 16 immune cells and 13 immune-related function activation (23). Thereafter, a comparison of TME scores was performed and expressions of immune checkpoints between different risk groups analyzed using “ggpubr” R package.

### Assessment of the potential therapeutic drugs

Based on the half-maximal inhibitory concentration (IC50) on Genomics of Drug Sensitivity in Cancer (GDSC) (https://www.cancerrxgene.org/) and clinical gene expression data, “pRRophetic” R package was used to predict the sensitivity of the potential therapeutic drugs for the AML patients in the two risk subgroups (24, 25).

### Statistical analysis

The R software (v4.1.3) was used to construct a risk model and for statistical analyses. Univariate and multivariate Cox regression analyses were performed to evaluate the independent prognostic value of clinical factors and risk model for AML patients. The Kaplan-Meier method with the two-side log-rank test was used to compare the OS time. Student’s t-test or Mann-Whitney test were used to determine the relationship between the risk score and clinical factors. For comparisons of immune cell infiltrations and immune scores between the two risk groups, the Mann-Whitney test was used. *p* < 0.05 was the threshold for significance.

## Results

### Identification of cuproptosis-related lncRNAs in AML patients

**Figure 1:**
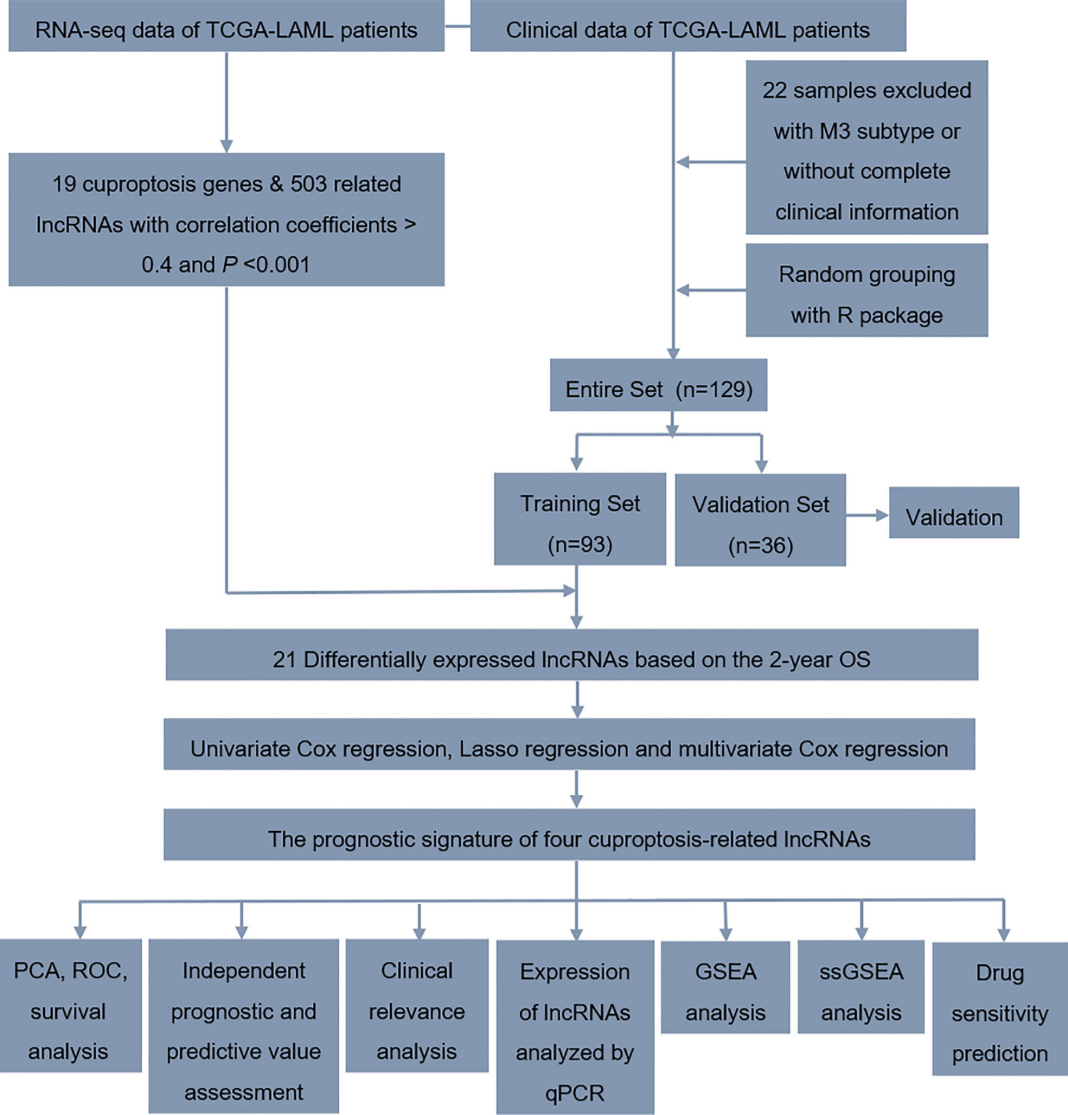
Workflow of the study design.

**Figure 2:**
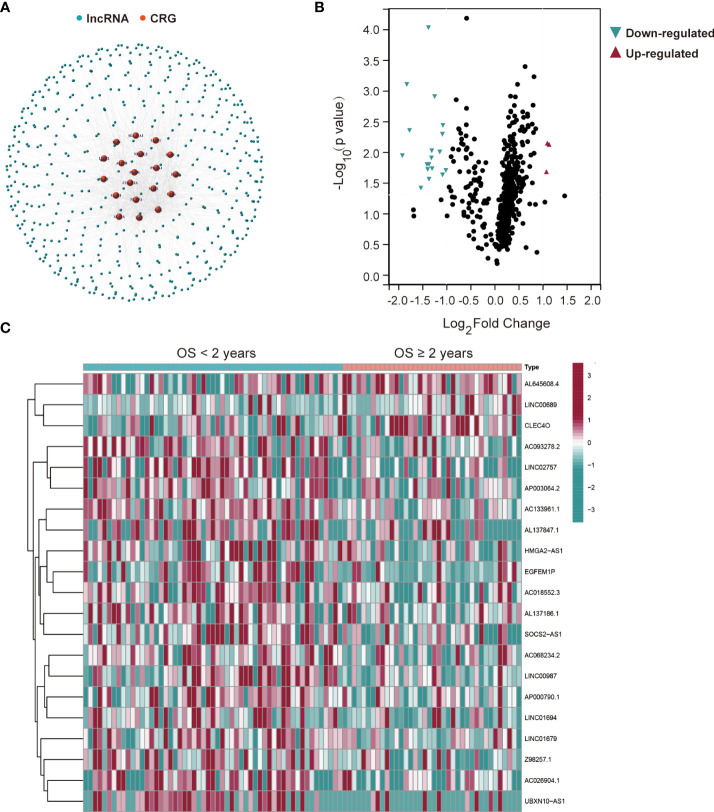
Twenty-one differentially expressed cuproptosis-related lncRNAs in AML patients from the TCGA database. **(A)** The network between 19 cuproptosis-related genes (CRGs) and 503 lncRNAs in AML patients (correlation coefficients > 0.4 and *p* < 0.001). **(B)** Differentially expressed lncRNAs for AML patients based on the OS < or ≥ 2 years. **(C)** A heatmap of the twenty-one differentially expressed lncRNAs.

The workflow of this study is shown in **Figure 1**. Mutant statuses of the 19 CRGs in AML patients from the TCGA database were analyzed by the cBioPortal database, which revealed that almost all CRGs had no significant genetic alterations (**Figure S1**). 503 lncRNAs that were associated with the 19 CRGs in AML samples from the TCGA database were obtained (**Figure 2A**). The correlations and regulation between CRGs and these lncRNAs are shown in **Table S4**. Next, 93 AML patients in the training set were assigned into two subgroups according to OS < 2 years (n = 55) and ≥ 2 years (n = 38) criteria. Twenty-one significantly differentially expressed lncRNAs were identified (**Figures 2B, C**).

### A cuproptosis-related lncRNA prognostic model for the AML patients

**Figure 3:**
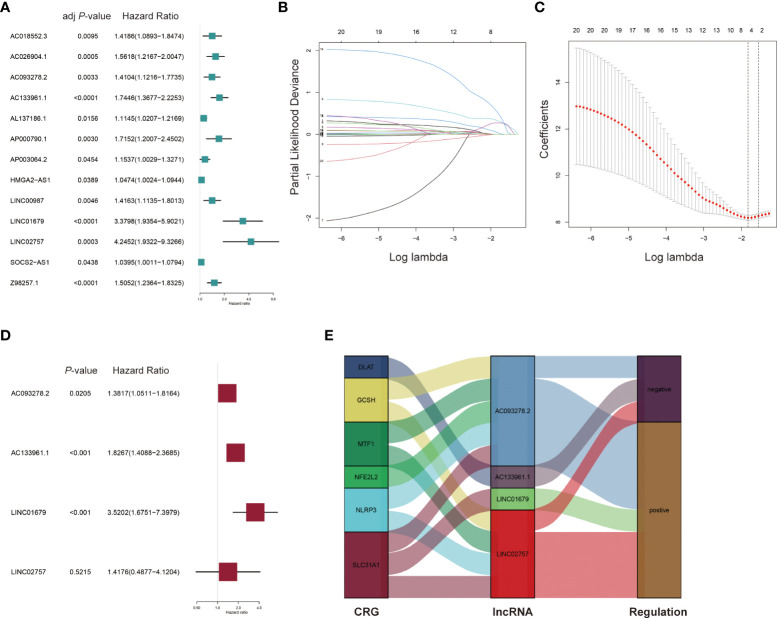
Construction of the prognostic cuproptosis-related lncRNA signature for AML patients in the training set. **(A)** Univariate Cox regression analysis for the prognostic cuproptosis-related lncRNAs. **(B, C)** Identification of prognostic cuproptosis-related lncRNAs in AML patients with 10-fold cross-validation for variable selection in LASSO (Least Absolute Shrinkage and Selection Operator) Cox regression. **(D)** Multivariate Cox regression analysis of the four cuproptosis-related lncRNAs for the prognosis of AML patients. **(E)** The Sankey diagram of cuproptosis-related genes (CRGs) and related lncRNAs.

Univariate Cox regression analysis was performed using the 21 differentially expressed lncRNAs in the training set and 13 lncRNAs (AC018552.3, AC026904.1, AC093278.2, AC133961.1, AL137186.1, AP000790.1, AP003064.2, HMGA2-AS1, LINC00987, LINC01679, LINC02757, SOCS2-AS1, Z98257.1) were found to be significantly associated with OS and an increased risk for AML (all adjusted *p*-value < 0.05 and HR > 1) (**Table 1** and **Figure 3A**). Then, a 4-lncRNA signature for prognosis was established based on LASSO regression (**Figures 3B, C**) and multivariate Cox regression (**Figure 3D**). We performed the multicollinearity test with all VIF < 2 (**Table S5**) and checked for violations of the proportional hazard (PH) hypothesis (**Table S6** and **Figure S2**). All covariates in the multivariate Cox regression satisfied the PH hypothesis and multicollinearity test. Risk Score = 0.3233 × AC093278.2_expression_ + 0.6025 × AC133961.1_expression_ + 1.2585 × LINC01679_expression_ + 0.3490 × LINC02757_expression_. Regulatory relationships between CRGs and the four lncRNAs are shown with a Sankey chart (**Figure 3E**).

**Table 1:** Univariate Cox regression of 13 lncRNAs for the OS of AML patients.

Ensemble Accession	lncRNA	HR (95%CI)	Adjusted *P-*value
ENSG00000261633	AC018552.3	1.149 (1.089-1.847)	0.009
ENSG00000253140	AC026904.1	1.562 (1.217-2.005)	< 0.001
ENSG00000261269	AC093278.2	1.410 (1.222-1.774)	0.003
ENSG00000251009	AC133961.1	1.745 (1.368-2.225)	< 0.001
ENSG00000229664	AL137186.1	1.115 (1.021-1.217)	0.016
ENSG00000214788	AP000790.1	1.715 (1.201-2.450)	0.003
ENSG00000255446	AP003064.2	1.154 (1.003-1.327)	0.045
ENSG00000197301	HMGA2-AS1	1.047 (1.002-1.094)	0.039
ENSG00000237248	LINC00987	1.416 (1.114-1.801)	0.005
ENSG00000237989	LINC01679	3.380 (1.935-5.902)	< 0.001
ENSG00000255363	LINC02757	4.245 (1.932-9.327)	< 0.001
ENSG00000246985	SOCS2-AS1	1.039 (1.001-1.079)	0.044
ENSG00000227066	Z98257.1	1.505 (1.236-1.833)	< 0.001

HR, Hazard Ratio.

### Performance of the signature for AML patients in the TCGA database

**Figure 4:**
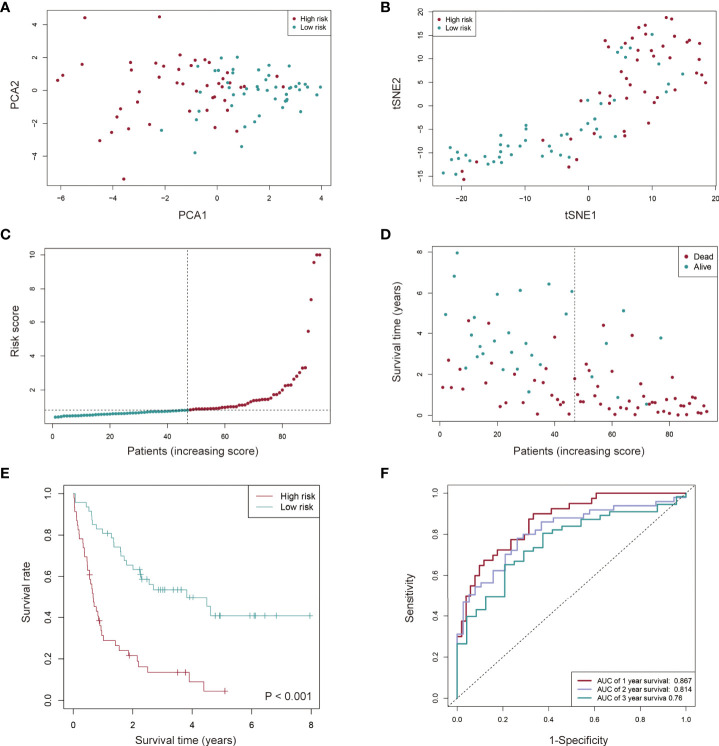
Prognostic value of the cuproptosis-related lncRNA signature in AML patients from the TCGA cohort. **(A, B)** AML patients in distinct risk groups could be separated into two clusters based on the PCA (Principal component analysis) **(A)** and t-distributed stochastic neighbor embedding (t-SNE) method **(B)**. **(C, D)** Distribution of the risk score **(C)** and survival time of each patient **(D)**. **(E)** Kaplan-Meier survival analysis of overall survival (OS) for AML patients with low- and high-risk groups. **(F)** The sensitivity and specificity of the prognostic model for AML patients assessed using the 1-, 2-, and 3-year ROC (receiver operating characteristic) curves and AUC (area under curve).

Based on the risk score, 93 AML patients were assigned into low- and high-risk groups (n = 47 and n = 46, respectively). Principal component analysis (PCA) and t-distributed Stochastic Neighbor Embedding (t-SNE) were performed. It was found that AML patients in distinct risk groups could be separated into two clusters (**Figures 4A, B**). Compared to the low-risk group, more death events and a shorter OS time were observed in the high-risk group (**Figures 4C-E**). Sensitivity and specificity of this novel prognostic model for AML patients were assessed using the receiver operating characteristic (ROC) method, and the area under the ROC curve (AUC) found to be 0.867, 0.814 and 0.760 for 1-, 2-, 3-year survival, respectively (**Figure 4F**), suggesting a high predictive power of the signature in the training set.

**Figure 5:**
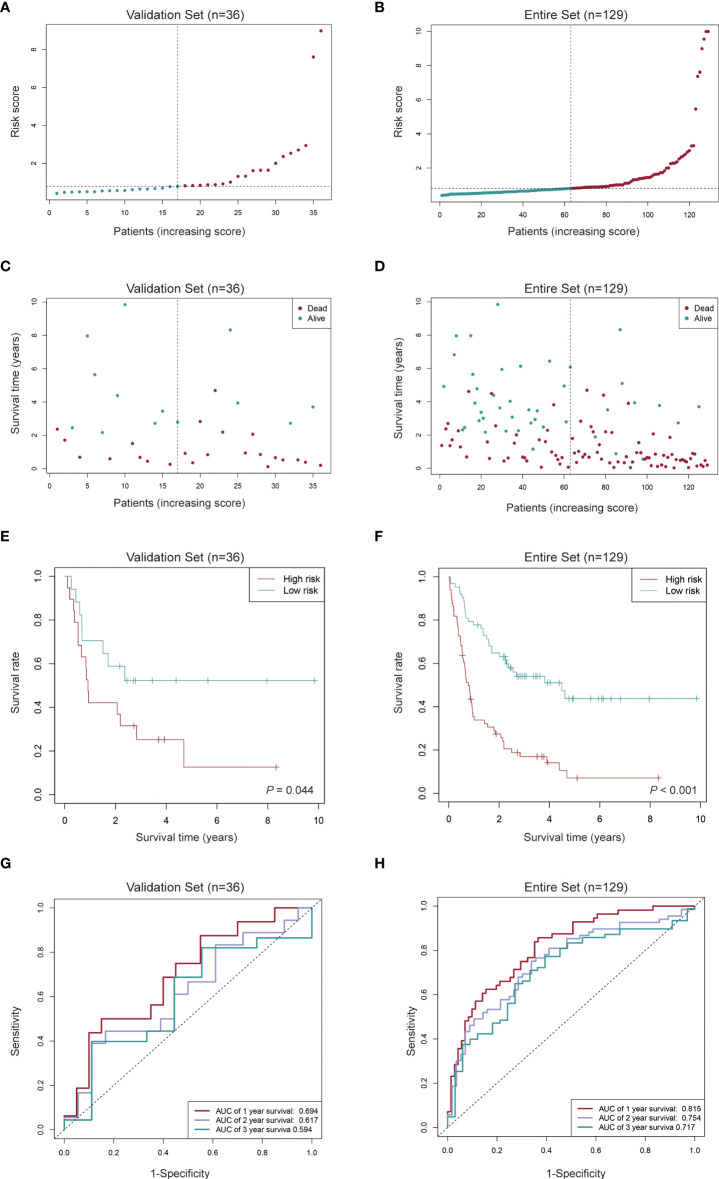
Evaluation of the cuproptosis-related lncRNA signature in the validation set and the entire set. **(A-D)** Distribution of the risk score and survival time of each patient in the validation set **(A, C)** and entire set **(B, D)**. **(E, F)** Kaplan-Meier curve analysis of the signature in the validation set **(E)** and entire set **(F)**. **(G, H)** ROC curves and their AUC values showed 1-, 2-, and 3-year predictions in the validation set **(G)** and entire set **(H)**.

We used the same algorithm to compute the risk scores in testing and entire sets. Findings from risk score distribution plot and scatter plot analyses were in accordance with those from the training set (**Figures 5A-D**). Kaplan-Meier curve analyses revealed that high-risk AML patients had worse survival outcomes (**Figures 5E, F**). Additionally, AUCs for 1-, 2- and 3-year OS were 0.694, 0.617 and 0.594 in the testing set (**Figure 5G**), and 0.815, 0.754 and 0.717 in the entire set (**Figure 5H**), respectively. These findings imply a good performance of the signature for prognostic prediction.

### Independent prognostic value of the cuproptosis-related lncRNA signature for AML patients

**Figure 6:**
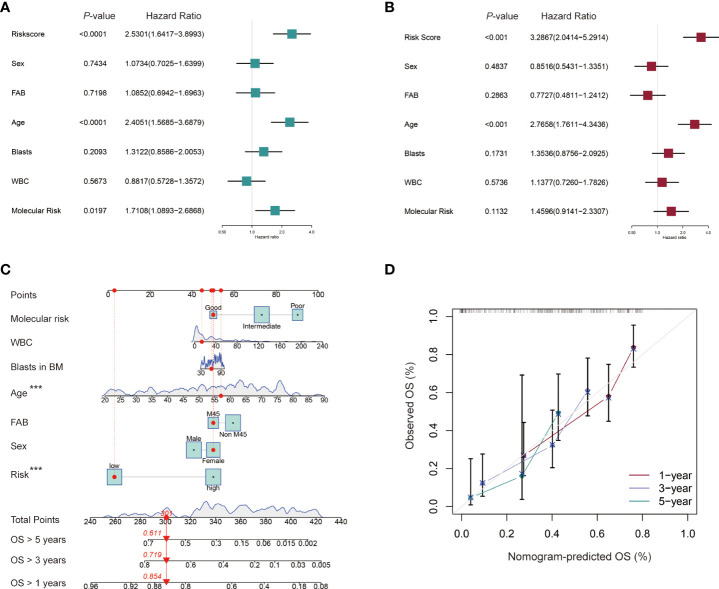
Independent prognostic role of the cuproptosis-related lncRNA signature for AML patients. **(A, B)** Univariate and multivariate Cox regression analysis of clinical factors including sex, French-American-British (FAB) subtype, age, blasts in bone marrow (BM), white blood counts (WBC), and molecular risk stratification and risk score for OS. **(C)** Nomogram that integrated the risk score and clinical factors predicts the probability of the 1-, 3-, and 5-year OS. **(D)** The calibration curves for 1-, 3-, and 5-year OS.

Univariate and multivariate Cox regression analyses were performed to identify the independent prognostic factors for AML patients in the TCGA cohort. Seven factors were considered in the analyses, including sex (Male vs. Female), FAB classification (M4/M5 vs. Non-M4/M5 subtype), age (≥ 60 vs. < 60 years old), blasts in BM (≥ 70% vs. < 70%), WBC counts (≥ 30 vs. < 30×10^9^/L), molecular risk stratification (Poor vs. Good/Intermediate) and the established lncRNA risk model (high- vs. low-risk group). Univariate Cox regression showed that age, molecular risk stratification and the lncRNA risk model were associated with worse prognostic outcomes (HR = 2.4051, 95% CI: 1.5685−3.6879; HR = 1.7108, 95% CI: 1.0893−2.6868; HR = 2.5301, 95% CI: 1.6471−3.8993 (**Figure 6A**). The multivariable Cox regression further established the independent prognostic value of the risk model (HR = 3.2867, 95% CI: 2.0414−5.2914) (**Figure 6B**). A nomogram for 1-, 3-, and 5-year OS was plotted based on the above 7 factors. The TCGA-AB-2846 sample was taken as an example for assessment of the nomogram. The patient was a 57 years old female with M4/M5 subtype, WBC counts 13.6 × 10^9^/L, 61% blasts in the bone marrow, good molecular risk group and belonging to the low-risk group. The patient had 301 as her risk score and showed a possibility of 85.4%, 71.9% and 61.1% for the OS > 1-, 3-, and 5-year, respectively (**Figure 6C**). Calibration curves analyses further confirmed the accuracy of this predictive model (**Figure 6D**).

### Association between the cuproptosis-related lncRNA signature and clinicopathological features of AML patients

**Figure 7:**
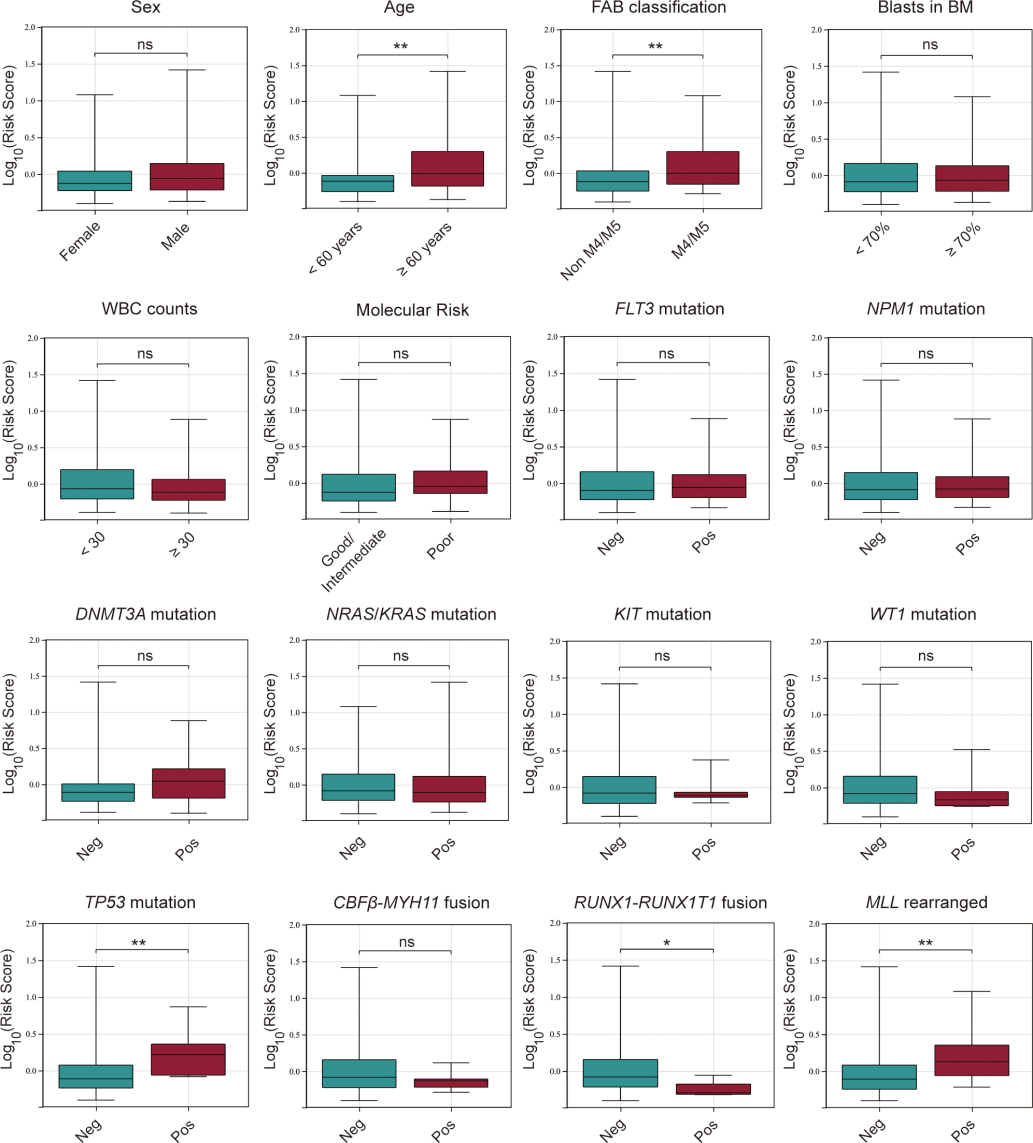
Association between the risk score and various clinicopathological factors in AML patients from the TCGA database. ns, no significance; *p < 0.05; **p < 0.01.

After constructing the cuproptosis-related lncRNA signature, we investigated its association with clinicopathological characteristics of AML patients, including sex, FAB classification, age, blasts in BM, WBC counts, molecular risk stratification, the status of gene mutations or rearrangements of *FLT3, NPM1, DNMT3A, NRAS*/*KRAS*, *TP53*, *WT1*, *KIT*, *MLL, CBFβ-MYH11, RUNX1-RUNX1T1.* It was found that AML patients with age > 60 years old, M4/M5 subtype, *TP53* mutation fusion and *MLL* rearranged had significantly higher risk scores while those with *RUNX1-RUNX1T1* had a lower risk score, and there were no significant differences between the risk score and other clinical variables (**Figure 7**).

### Expression levels of four cuproptosis-related lncRNAs investigated by qPCR in AML samples

**Figure 8:**
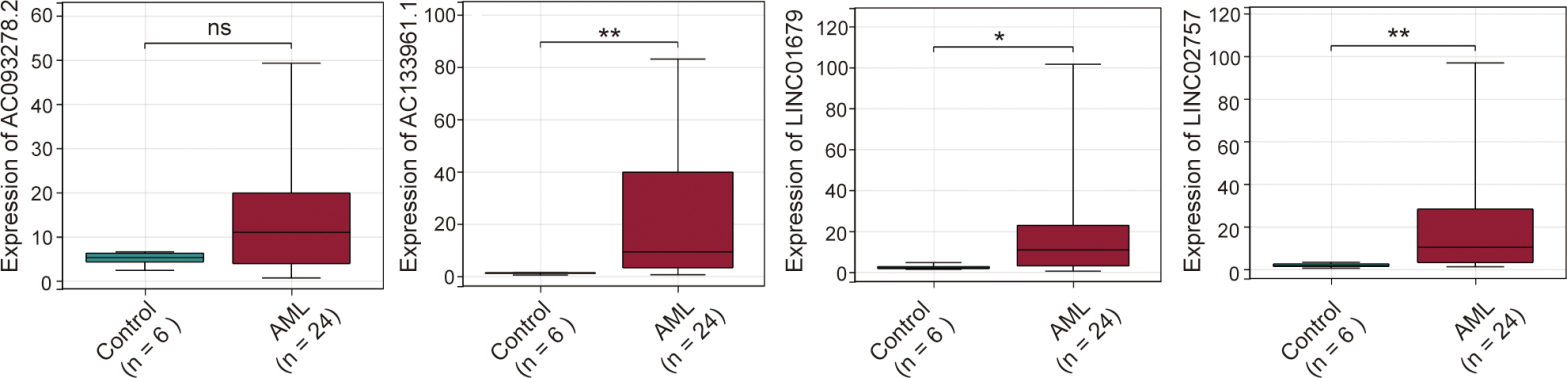
Expression levels of four cuproptosis-related lncRNAs in AML samples. ns:xFF1A;no significance; *p < 0.05; **p < 0.01.

qPCR was performed to examine the expressions of the four cuproptosis-related lncRNAs in 24 AML and 6 healthy control samples. Our results showed that the expression levels of the four lncRNAs were relatively higher in AML than that in controls. Besides AC093278.2, other three lncRNAs revealed a significant increase in AML patients (**Figure 8**).

### KEGG pathway analysis for high and low-risk AML patients

**Figure 9:**
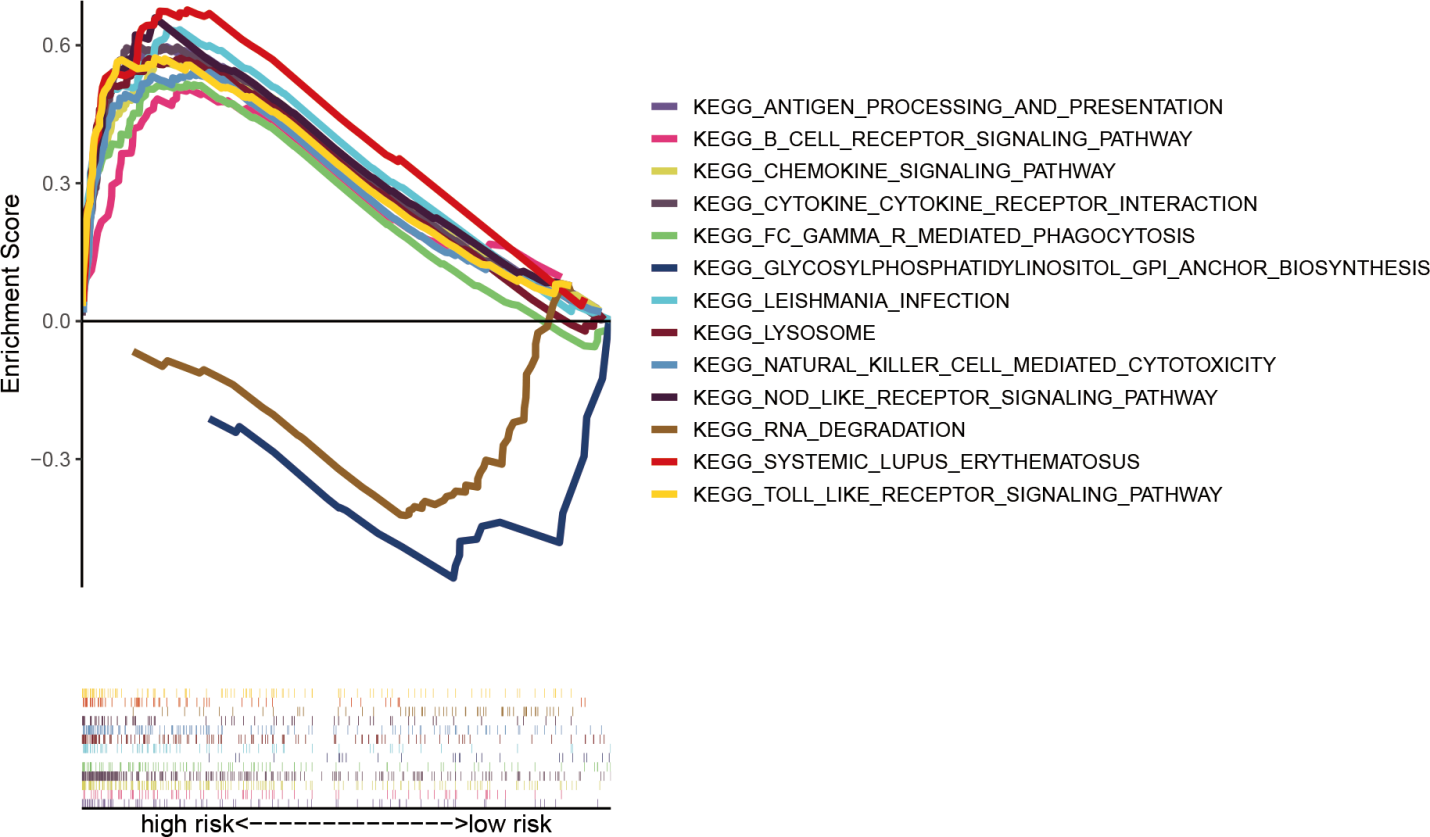
Significantly enriched KEGG pathways in AML patients with low- and high-risk groups analyzed using GSEA software.

To explore the functional mechanism between the two risk groups, enriched KEGG pathways were determined using the GSEA software. Fifty-six and two pathways were enriched in high- and low-risk groups, respectively (all |NES| > 1.5, NOM *p* < 0.05 and FDR < 0.25) (**Table S7**). Nearly all of the top eleven enriched pathways in the high-risk group were significantly correlated with the immune response (**Figure 9**), which provides the rationale for immune analysis in this risk model.

### Immune infiltration status and TME of AML patients in different risk groups

**Figure 10:**
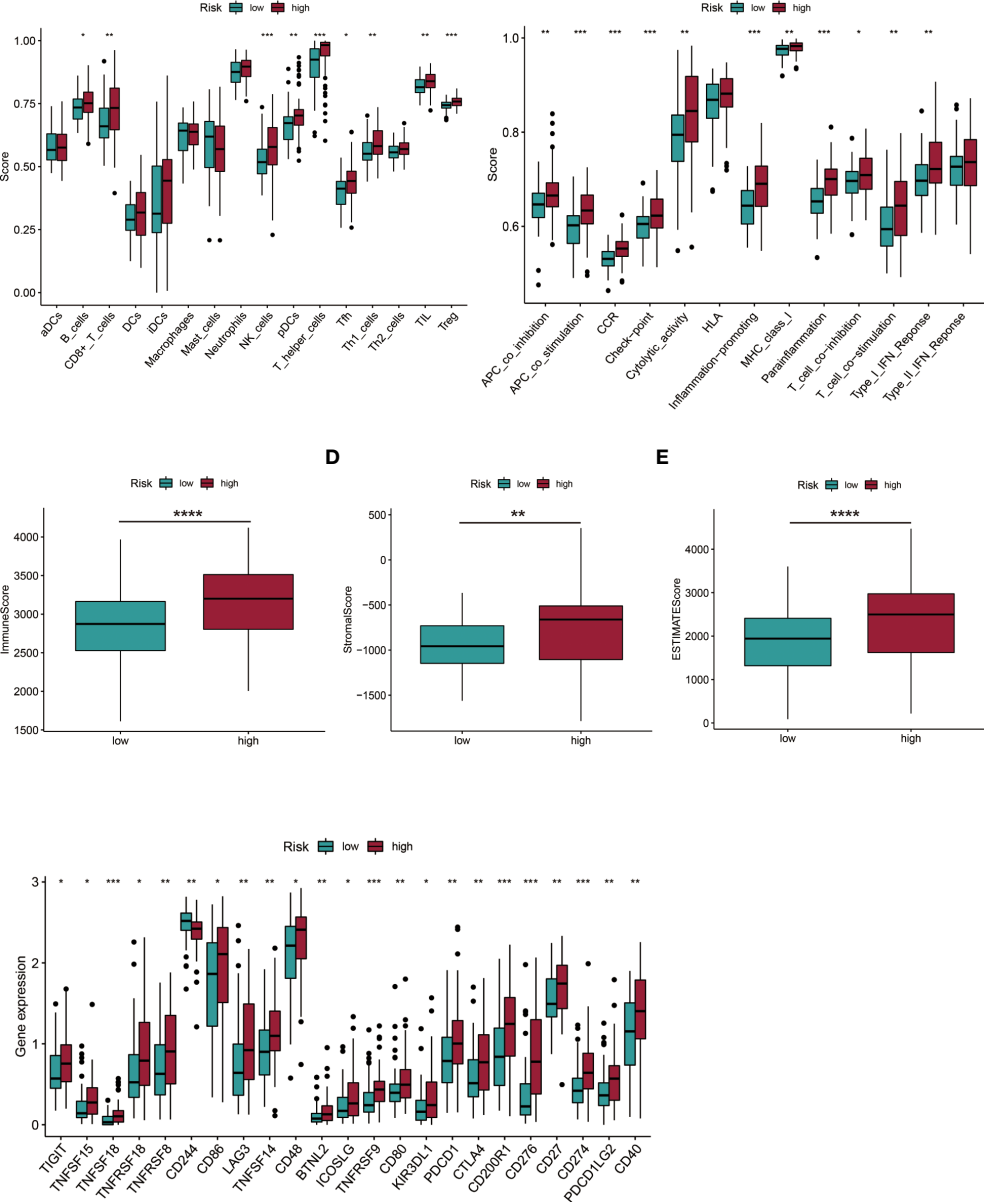
Investigation of immune cell infiltration and the tumor environment (TME). **(A, B)** Immune cell infiltration and immune-related function activation in the low- and high-risk group of AML patients. **(C-E)** Comparison of immune-related scores between low- and high-risk groups. **(F)** Twenty-three differentially expressed checkpoints between low- and high-risk groups. *P < 0.05; **P < 0.01; ***P < 0.005; ****P < 0.001.

The ssGSEA algorithm was used to assess the status and differences in immune cell infiltrations and immune-related function activation between AML patients in low- and high-risk groups. In **Figures 10A, B** cells, CD8^+^ T cells, natural killer (NK) cells, plasmacytoid DCs (pDCs), T helper cells, Th1 cells, tumor-infiltrating lymphocytes (TIL), and regulatory T cells (Tregs) were significantly upregulated in the high-risk group (all *p* < 0.05). APC co-inhibition and co-stimulation, CCR, checkpoint, cytolytic activities, inflammation promotion, MHC class I, para-inflammation, T cell co-inhibition and co-stimulation, and Type I IFN responses were highly increased in the high-risk group (all *p* < 0.05, **Figure 10B**). Immune and ESTIMATE scores revealed different TME for AML patients in high-risk and low-risk groups (**Figures 10C-E**). Elevated expressions of multiple immune checkpoints were prevalent in the high-risk group (**Figure 10F**).

### Prediction of potential therapeutic drugs for AML patients in different risk groups

**Figure 11:**
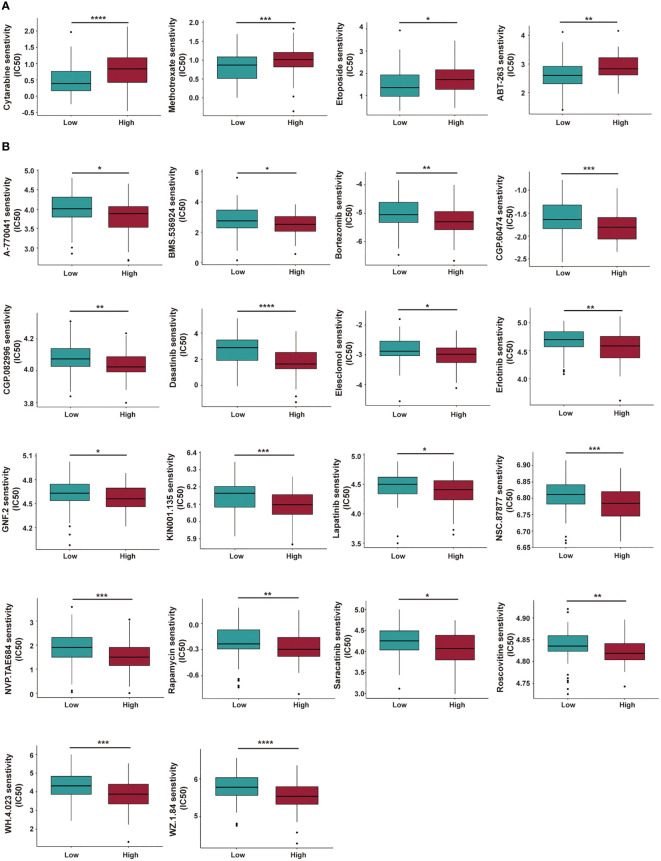
Investigation of chemotherapeutic and targeted therapy for AML patients with low- and high-risk groups. **(A, B)** The high-risk AML patients have a higher IC50 **(A)** and lower IC50 **(B)** for multiple potential drugs based on the GDSC database. IC50: Half-maximal Inhibitory Concentration. GDSC, Genomics of Drug Sensitivity in Cancer. *P < 0.05; **P < 0.01; ***P < 0.005; ****P < 0.001.

The sensitivity of various chemotherapeutic or targeted drugs for AML patients within different risk groups was predicted. In **Figure 11**, high-risk AML patients had a high IC50 for cytarabine, methotrexate, etoposide, and ABT-263, and a lower IC50 for rapamycin and bortezomib. The targets and involved pathways for these drugs are shown in **Table S8**.

## Discussion

As a newly defined type of programmed cell death, the exact role of cuproptosis in various cancer types has yet to be conclusively established; moreover, studies have not elucidated on the relationship between cuproptosis and lncRNA in AML. Therefore, we aimed at constructing a novel prognostic model that is based on cuproptosis-related lncRNAs for improved outcomes in AML patients.

In this study, CRGs were obtained from literatures and mutation analyses did not reveal significant genetic alterations for these genes in AML patients from the TCGA database. Next, 503 cuproptosis-related lncRNAs were identified using correlation analyses. To construct a cuproptosis-related prognostic model, 129 AML patients from the TCGA database were randomized into training and validation sets. Based on clinical experience and literatures, 93 AML patients in the training set were assigned into two groups using 2-year OS as the cutoff, and 21 differentially expressed lncRNAs were obtained. Out of the 21 lncRNAs, 13 were identified using univariate Cox regression to have a prognostic potential. To avoid overfitting, LASSO regression was performed to obtain a 4-cuproptosis-related lncRNA signature. The PCA, Kaplan-Meier survival and AUC analyses were performed to verify the distinguishing ability and accuracy of the established lncRNA signature. Then, validation and entire sets were used to confirm the prognostic value of the risk model. Moreover, multivariate Cox regression analyses were performed to confirm the independent prognostic value of the four lncRNA signatures for AML patients. Older patients, *TP53* mutation fusion or *MLL* rearranged, which are independent adverse prognostic factors for AML patients (2), had higher risk scores, and those with *RUNX1-RUNX1T1*, a favorable fusion gene, had lower risk scores. Furthermore, expressions of AC133961.1, LINC01679 and LINC02757 were found to be significantly increased in AML patients compared to healthy controls by qPCR assay.

Among the 4 cuproptosis-related lncRNA signatures, LINC01679 was initially identified in a 4 lncRNAs-based prognostic model for classification and prediction of survival in patients with prostate cancer (PCa) (26). A study (27) investigated the role of LINC01679 in PCa and found that patients with low expressions of LINC01679 had worse survival outcomes. Mechanistically, LINC01679, serving as a competitive endogenous RNA (ceRNA) inhibits PCa development and progression by regulating the miR-3150a-3p/SLC17A9 axis. Notably, LINC01679 was found to be a protective factor for PCa in both studies. In this study, LINC01679 was associated with increased risk for AML, suggesting that LINC01679 may have distinct roles in various cancer types. In previous studies, AC093278.2 was found to be an immune-related lncRNA for kidney renal clear cell carcinoma (28), while AC133961.1 was reported to be a ferroptosis-related lncRNA in AML with diagnostic and adverse prognostic roles (11). In addition, LINC02757 discovered in this study were not reported previously, of which the role needs to be further elucidated.

To investigate the functional mechanism of the constructed cuproptosis-related lncRNA signature for AML prognosis, KEGG pathway analysis was performed using the GSEA software. Multiple signaling pathways, especially immune-related processes, were found to be significantly enriched in the high-risk group. Previously, a variety of genes signatures associated with tumor immune microenvironment and immunotherapy response for pediatric or adult AML patients were established (29–33), and studies have reported on potential clinical benefits of immunotherapy against AML, including targeting CD33, CD123, and several immune checkpoint inhibitors (ICIs) (4, 34). In this study, we found high immune cell infiltrations and immune-related function activations in high-risk AML patients. The presence of T cells at the tumor site is critical for immune recognition and elimination of AML cells, and a higher percentage of CD8+ T cells in BM were predictive of responses to ICIs combined with a hypomethylating agent in AML patients (3, 35). The proportion of NK cells in the BM of AML patients effectively predicted their prognostic outcomes and a combination of NK cell-based immunotherapies with an MCL1 inhibitor showed synergistic anti-leukemia effects *in vitro* (36). Elevated pDC infiltrations are associated with immune escape and may be used for risk stratification for AML and to transdifferentiate leukemia in AML microenvironments (37, 38). Tregs were shown to be major contributors to defective immune responses, with increased Treg levels being associated with worse outcomes in AML (39, 40). Intriguingly, expressions of multiple well-known checkpoints were found to be significantly upregulated in high-risk AML patients. Programmed cell death 1 (PD-1), encoded by the PDCD1 gene, and its ligands PD-L1 (also known as CD274) and PD-L2 (also known as PDCD1LG2 or CD273), plays a crucial role in maintaining self-tolerance and are associated with immune escape in cancer by inhibiting the direct cytotoxic activities of effector CD8^+^ T cells on tumor cells (34). Elevated expressions of PD-1, PD-L1, and PD-L2 were associated with worse OS in AML patients (41), while the inhibition of the PD-1/PD-L1 pathway combined with hypomethylating agents or chemotherapy was found to be feasible and effective for newly diagnosed and refractory/relapsed (R/R) AML patients (3). CTLA-4, associated with the attenuation of T cell activation by preventing CD28 on T cells from binding its co-stimulatory counterparts (CD80 and CD86) on antigen-presenting cells (APCs) (34), demonstrated a modest efficacy for AML *in vitro* and *in vivo* upon blocking (4). Overall, the developed cuproptosis-related lncRNA signature elucidates on the relationship between cuproptosis and immunity as well as the rationale for the use of immunotherapy in AML patients.

Finally, drug sensitivities for AML patients in different risk groups were predicted. AML patients in the high-risk group showed a lower sensitivity to a range of anti-leukemia agents/drugs, including cytarabine, methotrexate, etoposide, and ABT-263 (a BCL-2 inhibitor, also called Navitoclax), while they responded better to several other drugs like rapamycin, bortezomib, Erlotinib, even though some of them are currently not in clinical use for treatment of AML. Based on our cuproptosis-related lncRNA risk model and the above findings, we concluded that a combination of immunotherapy with chemotherapy or other target inhibitors will provide a precise and personalized treatment strategy for AML patients.

This study has some limitations. First, we used the AML samples from the TCGA database and only conducted internal validation, since there was a lack of a suitable externally validated cohort to evaluate the effectiveness and reliability of the cuproptosis-related lncRNA signature. Moreover, we investigated the expressions of lncRNAs in the signature with a small sample size, and did not perform experiments to investigate the functions of the cuproptosis-related lncRNAs. Therefore, the established risk model established should be further tested by other researchers. Despite these limitations, this is the first study to construct a cuproptosis-related lncRNA signature for AML patients, and the risk model provides rational therapeutic strategies.

## Conclusion

We constructed a prognostic cuproptosis-related lncRNA signature for AML patients. The model provides an independent prognostic value, and novel insights into the potential therapeutic strategies, including immunotherapy for AML patients.

## Data availability statement

The datasets presented in this study can be found in online repositories. The names of the repository/repositories and accession number(s) can be found in the article/**Supplementary Material**.

## Ethics statement

The studies involving human participants were reviewed and approved by ethical committee of the West China Hospital of Sichuan University. The patients/participants provided their written informed consent to participate in this study. Written informed consent was obtained from the individual(s) for the publication of any potentially identifiable images or data included in this article.

## Author contributions

FJW conceived and designed this study, reviewed, and revised the manuscript. PL and JJL conducted the data analysis and wrote the original draft. FWen, YXC, ZYL, and JZ contributed in methodology and data collection. FWu, ZQL, and WLL performed qPCR and data analysis. All authors contributed to the article and approved the submitted version.

## Funding

This study was supported by the Natural Science Foundation of Hunan Province (2021JJ40501, 2020JJ8095) and the Health Commission Foundation of Hunan Province (202203044326, 202214014454).

## Acknowledgments

We are thankful to Li Wang, Ph.D. (from the Department of, Zhongshan University, Guangzhou, China) for providing guidance of data processing. We also thank Bullet Edits Limited for the linguistic editing and proofreading of the manuscript.

## Conflict of interest

The authors declare that the research was conducted in the absence of any commercial or financial relationships that could be construed as a potential conflict of interest.

## Publisher’s note

All claims expressed in this article are solely those of the authors and do not necessarily represent those of their affiliated organizations, or those of the publisher, the editors and the reviewers. Any product that may be evaluated in this article, or claim that may be made by its manufacturer, is not guaranteed or endorsed by the publisher.
