# Supplementary material for: A novel cuproptosis-related LncRNA signature: Prognostic and therapeutic value for acute myeloid leukemia
Source: Front Oncol. 2022 Oct 7;12:966920. doi: 10.3389/fonc.2022.966920 (PMC9585311; doi:10.3389/fonc.2022.966920)
Supplement: Supplementary file 1 [file DataSheet_1.docx]

**
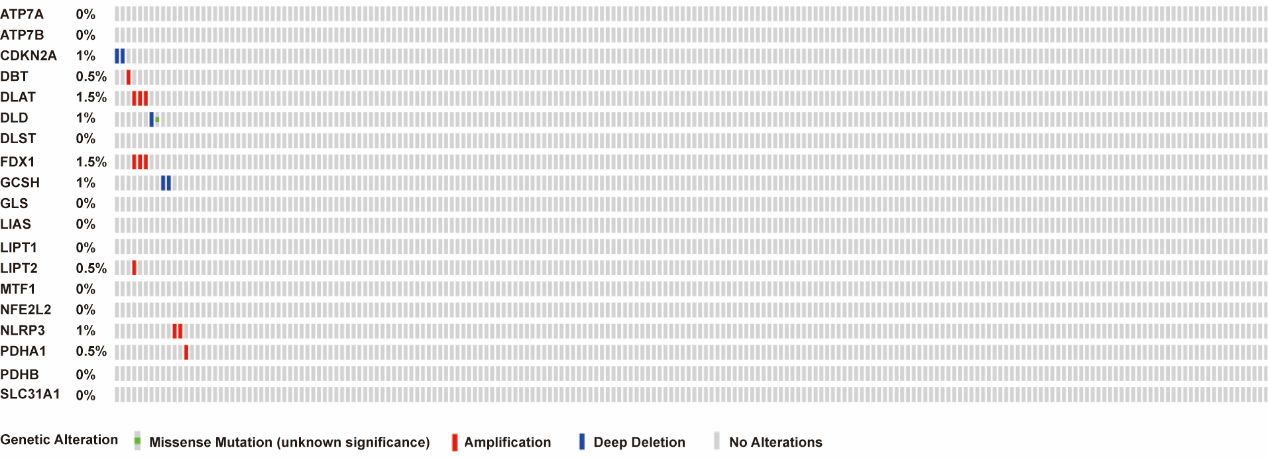
**

**Figure S1. Genetic alterations of 19 CRGs in AML patients from TCGA database.** CRGs: cuproptosis-related genes.


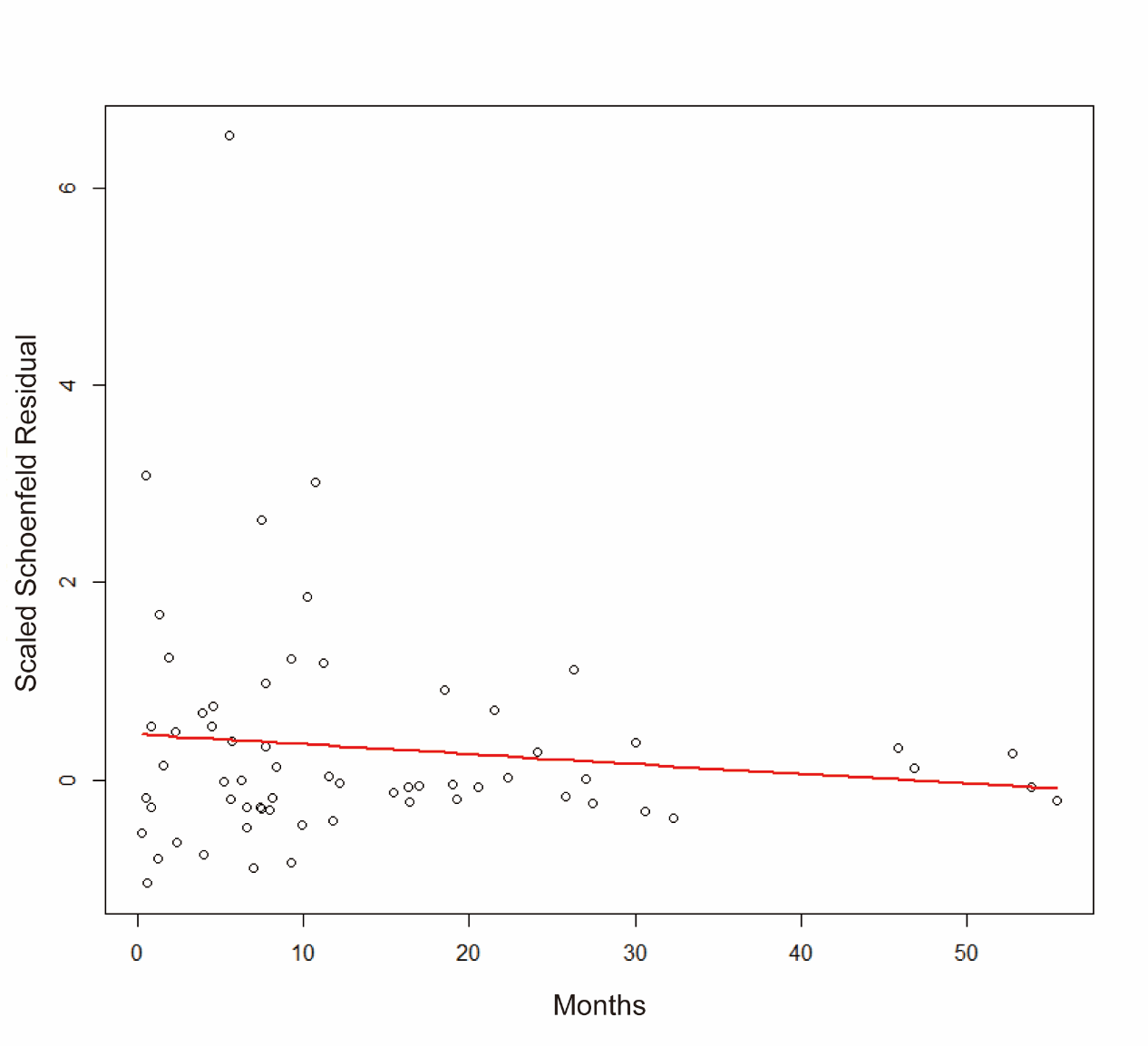


**Figure S2. Scaled Schoenfeld Residual test for the four lncRNAs in the signature**
