# Supplementary material for: A novel cuproptosis-related LncRNA signature: Prognostic and therapeutic value for acute myeloid leukemia
Source: Front Oncol. 2022 Oct 7;12:966920. doi: 10.3389/fonc.2022.966920 (PMC9585311; doi:10.3389/fonc.2022.966920)
Supplement: Supplementary file 2 [file DataSheet_2.docx]

| **Table S1. Clinical characteristics of AML patients in TCGA database** | | |
| --- | --- | --- |
| Characteristics | Number | Percentage (%) |
| Sex |  |  |
| Female | 58 | 44.96 |
| Male | 71 | 55.04 |
| Age |  |  |
| < 60 years old | 71 | 55.04 |
| ≥ 60 years old | 58 | 44.96 |
| Race |  |  |
| White | 94 | 72.87 |
| Others | 35 | 27.13 |
| FAB subtype classification |  |  |
| Non M4/M5 | 87 | 67.44 |
| M4/M5 | 42 | 32.56 |
| Blasts in BM (%) |  |  |
| < 70 | 61 | 47.29 |
| ≥ 70 | 68 | 52.71 |
| WBC counts (×10^9^/L) |  |  |
| < 30 | 76 | 58.91 |
| ≥ 30 | 53 | 41.09 |
| Molecular risk stratification |  |  |
| Good | 17 | 13.18 |
| Intermediate | 75 | 58.14 |
| Poor | 37 | 28.68 |
| Gene mutation |  |  |
| *FLT3* | 35 | 27.13 |
| *NPM1* | 35 | 27.13 |
| *DNMT3A* | 34 | 26.36 |
| *NRAS*/*KRAS* | 14 | 10.85 |
| *TP53* | 10 | 7.75 |
| *WT1* | 10 | 7.75 |
| *KIT* | 7 | 5.43 |
| Gene rearrangement |  |  |
| *CBFβ-MYH11* | 10 | 7.75 |
| *RUNX1-RUNX1T1* | 7 | 5.43 |
| *MLL* | 15 | 11.63 |
| OS |  |  |
| < 2 years | 73 | 56.59 |
| ≥ 2 years | 56 | 43.41 |
| Living status |  |  |
| Alive | 42 | 32.56 |
| Dead | 87 | 67.44 |
| FAB: French-American-British, BM: Bone Marrow, WBC: White Blood cells, OS: Overall Survival | | |

| **Table S2. Cuproptosis-related genes (CRGs)** | | | | | |  |  |  |  |
| --- | --- | --- | --- | --- | --- | --- | --- | --- | --- |
| ATP7A | DLD | | LIAS | | NLRP3 |  |  |  |  |
| ATP7B | DLST | | LIPT1 | | PDHA1 |  |  |  |  |
| CDKN2A | FDX1 | | LIPT2 | | PDHB |  |  |  |  |
| DBT | GCSH | | MTF1 | | SLC31A1 |  |  |  |  |
| DLAT | GLS | | NFE2L2 | |  |  |  |  |  |
| \| **Table S3. The primers sequences for qPCR** \| \| \| \| \| --- \| --- \| --- \| --- \| \| Gene \| \| Forward (5'-3') \| Reverse (5'-3') \| \| AC093278.2 \| GCATGACTGGCCTATGGTGA \| \| AGTCACACGTACACGACACC \| \| AC133961.1 \| AAGAAACCTCTCCGTGAAAAGC \| \| GCACCAGGCATGCAGAAAAC \| \| LINC01679 \| AAGACCTGCAGTCAATGGGG \| \| GGCGTTTGCAAAAGGGAGAG \| \| LINC02757 \| CGTCTCCACGGAGCATAGTC \| \| CTCCCAGCCTCTCCAAACAG \| \| GAPDH \| AATGAAGGGGTCATTGATGG \| \| AAGGTGAAGGTCGGAGTCAA \|   **Table S4. Correlation and regulation of CRGs and lncRNAs** | | | | | | | | |  |
| CRG | | lncRNA | | Correlation | | | *P*-value | Regulation |  |
| ATP7A | | AC000120.2 | | 0.449 | | | 0.001 | positive |  |
| ATP7A | | AC004492.1 | | 0.449 | | | 0.001 | positive |  |
| ATP7A | | AC004918.5 | | 0.490 | | | < 0.001 | positive |  |
| ATP7A | | AC005034.6 | | 0.479 | | | < 0.001 | positive |  |
| ATP7A | | AC006141.1 | | 0.479 | | | < 0.001 | positive |  |
| ATP7A | | AC006213.5 | | 0.490 | | | < 0.001 | positive |  |
| ATP7A | | AC008555.1 | | 0.542 | | | < 0.001 | positive |  |
| ATP7A | | AC009095.1 | | 0.472 | | | < 0.001 | positive |  |
| ATP7A | | AC009120.2 | | 0.488 | | | < 0.001 | positive |  |
| ATP7A | | AC009318.2 | | 0.543 | | | < 0.001 | positive |  |
| ATP7A | | AC010260.1 | | 0.543 | | | < 0.001 | positive |  |
| ATP7A | | AC010285.1 | | 0.525 | | | < 0.001 | positive |  |
| ATP7A | | AC010976.1 | | 0.460 | | | < 0.001 | positive |  |
| ATP7A | | AC011005.4 | | 0.536 | | | < 0.001 | positive |  |
| ATP7A | | AC012063.1 | | 0.546 | | | < 0.001 | positive |  |
| ATP7A | | AC015849.3 | | 0.648 | | | < 0.001 | positive |  |
| ATP7A | | AC015849.4 | | 0.478 | | | < 0.001 | positive |  |
| ATP7A | | AC018926.1 | | 0.433 | | | < 0.001 | positive |  |
| ATP7A | | AC020634.2 | | 0.488 | | | < 0.001 | positive |  |
| ATP7A | | AC021086.1 | | 0.474 | | | < 0.001 | positive |  |
| ATP7A | | AC021546.1 | | 0.541 | | | < 0.001 | positive |  |
| ATP7A | | AC022272.1 | | 0.503 | | | < 0.001 | positive |  |
| ATP7A | | AC022306.2 | | 0.523 | | | < 0.001 | positive |  |
| ATP7A | | AC022558.1 | | 0.620 | | | < 0.001 | positive |  |
| ATP7A | | AC022558.3 | | 0.518 | | | < 0.001 | positive |  |
| ATP7A | | AC024267.5 | | 0.448 | | | < 0.001 | positive |  |
| ATP7A | | AC027031.2 | | 0.466 | | | < 0.001 | positive |  |
| ATP7A | | AC048382.1 | | 0.530 | | | < 0.001 | positive |  |
| ATP7A | | AC055764.1 | | 0.465 | | | < 0.001 | positive |  |
| ATP7A | | AC067945.3 | | 0.445 | | | < 0.001 | positive |  |
| ATP7A | | AC068025.1 | | 0.557 | | | < 0.001 | positive |  |
| ATP7A | | AC068234.2 | | 0.464 | | | < 0.001 | positive |  |
| ATP7A | | AC073655.1 | | 0.467 | | | < 0.001 | positive |  |
| ATP7A | | AC073655.2 | | 0.469 | | | < 0.001 | positive |  |
| ATP7A | | AC079921.1 | | 0.498 | | | < 0.001 | positive |  |
| ATP7A | | AC084824.4 | | 0.586 | | | < 0.001 | positive |  |
| ATP7A | | AC090425.3 | | 0.493 | | | < 0.001 | positive |  |
| ATP7A | | AC090607.1 | | 0.494 | | | < 0.001 | positive |  |
| ATP7A | | AC092574.1 | | 0.517 | | | < 0.001 | positive |  |
| ATP7A | | AC092611.1 | | 0.436 | | | < 0.001 | positive |  |
| ATP7A | | AC096586.2 | | 0.545 | | | < 0.001 | positive |  |
| ATP7A | | AC103923.1 | | 0.487 | | | < 0.001 | positive |  |
| ATP7A | | AC104958.2 | | 0.593 | | | < 0.001 | positive |  |
| ATP7A | | AC110792.3 | | 0.433 | | | < 0.001 | positive |  |
| ATP7A | | AC120114.1 | | 0.432 | | | < 0.001 | positive |  |
| ATP7A | | AC123567.2 | | 0.497 | | | < 0.001 | positive |  |
| ATP7A | | AC129510.1 | | 0.513 | | | < 0.001 | positive |  |
| ATP7A | | AC129510.2 | | 0.618 | | | < 0.001 | positive |  |
| ATP7A | | AC130469.1 | | 0.491 | | | < 0.001 | positive |  |
| ATP7A | | AL080317.2 | | 0.474 | | | < 0.001 | positive |  |
| ATP7A | | AL138963.1 | | 0.453 | | | < 0.001 | positive |  |
| ATP7A | | AL157400.4 | | 0.436 | | | < 0.001 | positive |  |
| ATP7A | | AL161756.1 | | 0.480 | | | < 0.001 | positive |  |
| ATP7A | | AL354696.2 | | 0.618 | | | < 0.001 | positive |  |
| ATP7A | | AL365361.1 | | 0.434 | | | < 0.001 | positive |  |
| ATP7A | | AL590428.1 | | 0.435 | | | < 0.001 | positive |  |
| ATP7A | | AL590652.1 | | 0.476 | | | < 0.001 | positive |  |
| ATP7A | | ANKRD33B-AS1 | | 0.584 | | | < 0.001 | positive |  |
| ATP7A | | AP000695.1 | | 0.443 | | | < 0.001 | positive |  |
| ATP7A | | AP001429.1 | | 0.449 | | | < 0.001 | positive |  |
| ATP7A | | ARPP21-AS1 | | 0.506 | | | < 0.001 | positive |  |
| ATP7A | | CTD-2245F17.3 | | 0.466 | | | < 0.001 | positive |  |
| ATP7A | | ENTPD1-AS1 | | 0.473 | | | < 0.001 | positive |  |
| ATP7A | | GTF2IP4 | | 0.513 | | | < 0.001 | positive |  |
| ATP7A | | GUSBP5 | | 0.534 | | | < 0.001 | positive |  |
| ATP7A | | HLA-F-AS1 | | 0.492 | | | < 0.001 | positive |  |
| ATP7A | | KIZ-AS1 | | 0.514 | | | < 0.001 | positive |  |
| ATP7A | | LINC00624 | | 0.484 | | | < 0.001 | positive |  |
| ATP7A | | LINC00674 | | 0.534 | | | < 0.001 | positive |  |
| ATP7A | | LINC00987 | | 0.430 | | | < 0.001 | positive |  |
| ATP7A | | LINC01088 | | 0.430 | | | < 0.001 | positive |  |
| ATP7A | | LINC01359 | | 0.533 | | | < 0.001 | positive |  |
| ATP7A | | LINC01801 | | 0.487 | | | < 0.001 | positive |  |
| ATP7A | | MYLK-AS2 | | 0.549 | | | < 0.001 | positive |  |
| ATP7A | | NADK2-AS1 | | 0.521 | | | < 0.001 | positive |  |
| ATP7A | | NARF-IT1 | | 0.428 | | | < 0.001 | positive |  |
| ATP7A | | NBPF25P | | 0.443 | | | < 0.001 | positive |  |
| ATP7A | | NNT-AS1 | | 0.526 | | | < 0.001 | positive |  |
| ATP7A | | NORAD | | 0.497 | | | < 0.001 | positive |  |
| ATP7A | | RP11-655M14.13 | | 0.476 | | | < 0.001 | positive |  |
| ATP7A | | SIDT1-AS1 | | 0.638 | | | < 0.001 | positive |  |
| ATP7A | | SRD5A3-AS1 | | 0.468 | | | < 0.001 | positive |  |
| ATP7A | | STARD4-AS1 | | 0.451 | | | < 0.001 | positive |  |
| ATP7A | | VAC14-AS1 | | 0.434 | | | < 0.001 | positive |  |
| ATP7A | | Z99572.1 | | 0.510 | | | < 0.001 | positive |  |
| ATP7A | | ZBED3-AS1 | | 0.490 | | | < 0.001 | positive |  |
| ATP7A | | ZNF451-AS1 | | 0.552 | | | < 0.001 | positive |  |
| ATP7B | | AC018552.3 | | 0.658 | | | < 0.001 | positive |  |
| ATP7B | | AC021086.1 | | 0.442 | | | < 0.001 | positive |  |
| ATP7B | | AC022447.6 | | 0.642 | | | < 0.001 | positive |  |
| ATP7B | | AC068234.2 | | 0.456 | | | < 0.001 | positive |  |
| ATP7B | | AC096677.1 | | 0.439 | | | < 0.001 | positive |  |
| ATP7B | | AC104958.2 | | 0.566 | | | < 0.001 | positive |  |
| ATP7B | | AC116025.2 | | 0.449 | | | < 0.001 | positive |  |
| ATP7B | | AL133215.2 | | 0.434 | | | < 0.001 | positive |  |
| ATP7B | | AL162727.2 | | 0.562 | | | < 0.001 | positive |  |
| ATP7B | | AL359094.1 | | 0.453 | | | < 0.001 | positive |  |
| ATP7B | | AL590428.1 | | 0.511 | | | < 0.001 | positive |  |
| ATP7B | | AL683807.1 | | 0.436 | | | < 0.001 | positive |  |
| ATP7B | | AP001107.9 | | 0.519 | | | < 0.001 | positive |  |
| ATP7B | | CHRM3-AS2 | | 0.459 | | | < 0.001 | positive |  |
| ATP7B | | CYTOR | | 0.467 | | | < 0.001 | positive |  |
| ATP7B | | EGFEM1P | | 0.482 | | | < 0.001 | positive |  |
| ATP7B | | GUSBP5 | | 0.586 | | | < 0.001 | positive |  |
| ATP7B | | HCP5 | | 0.526 | | | < 0.001 | positive |  |
| ATP7B | | LINC01218 | | 0.454 | | | < 0.001 | positive |  |
| ATP7B | | LINC01694 | | 0.546 | | | < 0.001 | positive |  |
| ATP7B | | LOXL1-AS1 | | 0.642 | | | < 0.001 | positive |  |
| ATP7B | | LRRC37A6P | | 0.524 | | | < 0.001 | positive |  |
| ATP7B | | MIR34AHG | | 0.429 | | | < 0.001 | positive |  |
| ATP7B | | RP11-524D16_A.3 | | 0.586 | | | < 0.001 | positive |  |
| ATP7B | | RPL10P19 | | 0.531 | | | < 0.001 | positive |  |
| ATP7B | | SDHAP3 | | 0.490 | | | < 0.001 | positive |  |
| ATP7B | | SOCS2-AS1 | | 0.578 | | | < 0.001 | positive |  |
| ATP7B | | TTC28-AS1 | | 0.493 | | | < 0.001 | positive |  |
| ATP7B | | UBXN10-AS1 | | 0.616 | | | < 0.001 | positive |  |
| ATP7B | | Z99572.1 | | 0.482 | | | < 0.001 | positive |  |
| CDKN2A | | AC091151.1 | | 0.589 | | | < 0.001 | positive |  |
| CDKN2A | | AC111000.4 | | 0.586 | | | < 0.001 | positive |  |
| CDKN2A | | AC113414.1 | | 0.431 | | | < 0.001 | positive |  |
| CDKN2A | | AL391121.1 | | 0.476 | | | < 0.001 | positive |  |
| CDKN2A | | UCA1 | | 0.479 | | | < 0.001 | positive |  |
| DBT | | AC000120.2 | | 0.489 | | | < 0.001 | positive |  |
| DBT | | AC002553.2 | | 0.473 | | | < 0.001 | positive |  |
| DBT | | AC004918.5 | | 0.496 | | | < 0.001 | positive |  |
| DBT | | AC005034.6 | | 0.668 | | | < 0.001 | positive |  |
| DBT | | AC005072.1 | | 0.458 | | | < 0.001 | positive |  |
| DBT | | AC006116.10 | | 0.501 | | | < 0.001 | positive |  |
| DBT | | AC006116.7 | | 0.532 | | | < 0.001 | positive |  |
| DBT | | AC006141.1 | | 0.609 | | | < 0.001 | positive |  |
| DBT | | AC006213.5 | | 0.667 | | | < 0.001 | positive |  |
| DBT | | AC007996.1 | | 0.644 | | | < 0.001 | positive |  |
| DBT | | AC008395.1 | | 0.574 | | | < 0.001 | positive |  |
| DBT | | AC008555.1 | | 0.540 | | | < 0.001 | positive |  |
| DBT | | AC008738.2 | | 0.440 | | | < 0.001 | positive |  |
| DBT | | AC008770.3 | | 0.520 | | | < 0.001 | positive |  |
| DBT | | AC009095.1 | | 0.491 | | | < 0.001 | positive |  |
| DBT | | AC009120.2 | | 0.671 | | | < 0.001 | positive |  |
| DBT | | AC009120.3 | | 0.486 | | | < 0.001 | positive |  |
| DBT | | AC009283.1 | | 0.519 | | | < 0.001 | positive |  |
| DBT | | AC009318.2 | | 0.445 | | | < 0.001 | positive |  |
| DBT | | AC009902.3 | | 0.437 | | | < 0.001 | positive |  |
| DBT | | AC010247.2 | | -0.514 | | | < 0.001 | negative |  |
| DBT | | AC010260.1 | | 0.442 | | | < 0.001 | positive |  |
| DBT | | AC010976.1 | | 0.562 | | | < 0.001 | positive |  |
| DBT | | AC011405.1 | | 0.447 | | | < 0.001 | positive |  |
| DBT | | AC011447.3 | | 0.626 | | | < 0.001 | positive |  |
| DBT | | AC011465.1 | | 0.449 | | | < 0.001 | positive |  |
| DBT | | AC011466.1 | | 0.478 | | | < 0.001 | positive |  |
| DBT | | AC011477.1 | | 0.647 | | | < 0.001 | positive |  |
| DBT | | AC011477.3 | | 0.528 | | | < 0.001 | positive |  |
| DBT | | AC012063.1 | | 0.559 | | | < 0.001 | positive |  |
| DBT | | AC015849.3 | | 0.667 | | | < 0.001 | positive |  |
| DBT | | AC015849.4 | | 0.609 | | | < 0.001 | positive |  |
| DBT | | AC018647.2 | | 0.621 | | | < 0.001 | positive |  |
| DBT | | AC018878.1 | | 0.483 | | | < 0.001 | positive |  |
| DBT | | AC018926.1 | | 0.477 | | | < 0.001 | positive |  |
| DBT | | AC020661.3 | | 0.566 | | | < 0.001 | positive |  |
| DBT | | AC020915.1 | | 0.442 | | | < 0.001 | positive |  |
| DBT | | AC021086.1 | | 0.494 | | | < 0.001 | positive |  |
| DBT | | AC021224.1 | | 0.485 | | | < 0.001 | positive |  |
| DBT | | AC021237.1 | | 0.463 | | | < 0.001 | positive |  |
| DBT | | AC021422.2 | | 0.501 | | | < 0.001 | positive |  |
| DBT | | AC021546.1 | | 0.443 | | | < 0.001 | positive |  |
| DBT | | AC022001.2 | | 0.459 | | | < 0.001 | positive |  |
| DBT | | AC022272.1 | | 0.519 | | | < 0.001 | positive |  |
| DBT | | AC022306.2 | | 0.707 | | | < 0.001 | positive |  |
| DBT | | AC022558.1 | | 0.566 | | | < 0.001 | positive |  |
| DBT | | AC022558.3 | | 0.551 | | | < 0.001 | positive |  |
| DBT | | AC022726.1 | | 0.451 | | | < 0.001 | positive |  |
| DBT | | AC023389.2 | | 0.458 | | | < 0.001 | positive |  |
| DBT | | AC024896.1 | | 0.633 | | | < 0.001 | positive |  |
| DBT | | AC024940.6 | | 0.524 | | | < 0.001 | positive |  |
| DBT | | AC025430.1 | | 0.478 | | | < 0.001 | positive |  |
| DBT | | AC025682.2 | | 0.586 | | | < 0.001 | positive |  |
| DBT | | AC026356.2 | | 0.589 | | | < 0.001 | positive |  |
| DBT | | AC026979.3 | | 0.480 | | | < 0.001 | positive |  |
| DBT | | AC034102.8 | | 0.522 | | | < 0.001 | positive |  |
| DBT | | AC046134.2 | | 0.569 | | | < 0.001 | positive |  |
| DBT | | AC046158.2 | | 0.531 | | | < 0.001 | positive |  |
| DBT | | AC048351.2 | | 0.556 | | | < 0.001 | positive |  |
| DBT | | AC048382.1 | | 0.499 | | | < 0.001 | positive |  |
| DBT | | AC055764.1 | | 0.553 | | | < 0.001 | positive |  |
| DBT | | AC055855.2 | | 0.640 | | | < 0.001 | positive |  |
| DBT | | AC068025.1 | | 0.672 | | | < 0.001 | positive |  |
| DBT | | AC073487.1 | | 0.488 | | | < 0.001 | positive |  |
| DBT | | AC078785.2 | | 0.544 | | | < 0.001 | positive |  |
| DBT | | AC078795.1 | | 0.461 | | | < 0.001 | positive |  |
| DBT | | AC083798.2 | | 0.476 | | | < 0.001 | positive |  |
| DBT | | AC083806.2 | | 0.563 | | | < 0.001 | positive |  |
| DBT | | AC084824.4 | | 0.703 | | | < 0.001 | positive |  |
| DBT | | AC087273.2 | | -0.473 | | | < 0.001 | negative |  |
| DBT | | AC090425.3 | | 0.638 | | | < 0.001 | positive |  |
| DBT | | AC090912.2 | | 0.490 | | | < 0.001 | positive |  |
| DBT | | AC091117.3 | | 0.478 | | | < 0.001 | positive |  |
| DBT | | AC091563.1 | | 0.446 | | | < 0.001 | positive |  |
| DBT | | AC092279.2 | | 0.530 | | | < 0.001 | positive |  |
| DBT | | AC092574.1 | | 0.693 | | | < 0.001 | positive |  |
| DBT | | AC092611.1 | | 0.531 | | | < 0.001 | positive |  |
| DBT | | AC093227.1 | | 0.530 | | | < 0.001 | positive |  |
| DBT | | AC096586.2 | | 0.591 | | | < 0.001 | positive |  |
| DBT | | AC097376.2 | | 0.455 | | | < 0.001 | positive |  |
| DBT | | AC097376.3 | | 0.448 | | | < 0.001 | positive |  |
| DBT | | AC103923.1 | | 0.500 | | | < 0.001 | positive |  |
| DBT | | AC104113.1 | | 0.537 | | | < 0.001 | positive |  |
| DBT | | AC104982.2 | | 0.445 | | | < 0.001 | positive |  |
| DBT | | AC109361.1 | | 0.575 | | | < 0.001 | positive |  |
| DBT | | AC110769.2 | | 0.456 | | | < 0.001 | positive |  |
| DBT | | AC110792.3 | | 0.475 | | | < 0.001 | positive |  |
| DBT | | AC112512.1 | | 0.462 | | | < 0.001 | positive |  |
| DBT | | AC127024.3 | | 0.430 | | | < 0.001 | positive |  |
| DBT | | AC127024.4 | | 0.577 | | | < 0.001 | positive |  |
| DBT | | AC127024.5 | | 0.492 | | | < 0.001 | positive |  |
| DBT | | AC129510.1 | | 0.464 | | | < 0.001 | positive |  |
| DBT | | AC129510.2 | | 0.511 | | | < 0.001 | positive |  |
| DBT | | AC130324.1 | | 0.488 | | | < 0.001 | positive |  |
| DBT | | AC138207.6 | | 0.467 | | | < 0.001 | positive |  |
| DBT | | AC139887.2 | | 0.458 | | | < 0.001 | positive |  |
| DBT | | AC141002.1 | | 0.447 | | | < 0.001 | positive |  |
| DBT | | AC211433.1 | | 0.467 | | | < 0.001 | positive |  |
| DBT | | AC211486.1 | | 0.585 | | | < 0.001 | positive |  |
| DBT | | AC244093.4 | | 0.473 | | | < 0.001 | positive |  |
| DBT | | AL031716.1 | | 0.541 | | | < 0.001 | positive |  |
| DBT | | AL080317.2 | | 0.657 | | | < 0.001 | positive |  |
| DBT | | AL121759.1 | | 0.493 | | | < 0.001 | positive |  |
| DBT | | AL132780.2 | | 0.457 | | | < 0.001 | positive |  |
| DBT | | AL133268.3 | | 0.491 | | | < 0.001 | positive |  |
| DBT | | AL138963.1 | | 0.477 | | | < 0.001 | positive |  |
| DBT | | AL139246.5 | | -0.470 | | | < 0.001 | negative |  |
| DBT | | AL139353.2 | | 0.646 | | | < 0.001 | positive |  |
| DBT | | AL157400.4 | | 0.538 | | | < 0.001 | positive |  |
| DBT | | AL158163.2 | | 0.530 | | | < 0.001 | positive |  |
| DBT | | AL161644.1 | | 0.433 | | | < 0.001 | positive |  |
| DBT | | AL161756.1 | | 0.612 | | | < 0.001 | positive |  |
| DBT | | AL353743.1 | | 0.514 | | | < 0.001 | positive |  |
| DBT | | AL354696.2 | | 0.759 | | | < 0.001 | positive |  |
| DBT | | AL354989.1 | | 0.555 | | | < 0.001 | positive |  |
| DBT | | AL356599.1 | | 0.527 | | | < 0.001 | positive |  |
| DBT | | AL359715.1 | | 0.541 | | | < 0.001 | positive |  |
| DBT | | AL359715.3 | | 0.541 | | | < 0.001 | positive |  |
| DBT | | AL391834.1 | | 0.517 | | | < 0.001 | positive |  |
| DBT | | AL442071.1 | | 0.485 | | | < 0.001 | positive |  |
| DBT | | AL442125.1 | | 0.465 | | | < 0.001 | positive |  |
| DBT | | AL512413.1 | | 0.478 | | | < 0.001 | positive |  |
| DBT | | AL590652.1 | | 0.614 | | | < 0.001 | positive |  |
| DBT | | AL591167.1 | | 0.476 | | | < 0.001 | positive |  |
| DBT | | ALG9-IT1 | | 0.484 | | | < 0.001 | positive |  |
| DBT | | ANKRD33B-AS1 | | 0.491 | | | < 0.001 | positive |  |
| DBT | | AP001429.1 | | 0.515 | | | < 0.001 | positive |  |
| DBT | | AP001542.3 | | 0.458 | | | < 0.001 | positive |  |
| DBT | | AP002907.1 | | 0.451 | | | < 0.001 | positive |  |
| DBT | | AP005432.1 | | 0.508 | | | < 0.001 | positive |  |
| DBT | | ASH1L-IT1 | | 0.531 | | | < 0.001 | positive |  |
| DBT | | BX470102.1 | | -0.505 | | | < 0.001 | negative |  |
| DBT | | CASC15 | | 0.486 | | | < 0.001 | positive |  |
| DBT | | CCT6P1 | | 0.480 | | | < 0.001 | positive |  |
| DBT | | CCT6P3 | | 0.473 | | | < 0.001 | positive |  |
| DBT | | DNAJC3-DT | | 0.569 | | | < 0.001 | positive |  |
| DBT | | E2F3-IT1 | | 0.439 | | | < 0.001 | positive |  |
| DBT | | EHMT2-AS1 | | 0.483 | | | < 0.001 | positive |  |
| DBT | | ENTPD1-AS1 | | 0.459 | | | < 0.001 | positive |  |
| DBT | | ERVH48-1 | | 0.479 | | | < 0.001 | positive |  |
| DBT | | FTX | | 0.539 | | | < 0.001 | positive |  |
| DBT | | GUSBP5 | | 0.518 | | | < 0.001 | positive |  |
| DBT | | INTS4P2 | | 0.435 | | | < 0.001 | positive |  |
| DBT | | IPO5P1 | | 0.505 | | | < 0.001 | positive |  |
| DBT | | KB-1674E1.2 | | 0.560 | | | < 0.001 | positive |  |
| DBT | | KIZ-AS1 | | 0.688 | | | < 0.001 | positive |  |
| DBT | | LINC00539 | | 0.532 | | | < 0.001 | positive |  |
| DBT | | LINC00624 | | 0.606 | | | < 0.001 | positive |  |
| DBT | | LINC00630 | | 0.582 | | | < 0.001 | positive |  |
| DBT | | LINC00674 | | 0.490 | | | < 0.001 | positive |  |
| DBT | | LINC01353 | | -0.489 | | | < 0.001 | negative |  |
| DBT | | LINC01359 | | 0.528 | | | < 0.001 | positive |  |
| DBT | | LINC01801 | | 0.486 | | | < 0.001 | positive |  |
| DBT | | LINC02175 | | 0.534 | | | < 0.001 | positive |  |
| DBT | | LINC02626 | | 0.444 | | | < 0.001 | positive |  |
| DBT | | LRRC37BP1 | | 0.690 | | | < 0.001 | positive |  |
| DBT | | MFSD13B | | 0.695 | | | < 0.001 | positive |  |
| DBT | | MIPEPP3 | | 0.513 | | | < 0.001 | positive |  |
| DBT | | MIR133A1HG | | 0.458 | | | < 0.001 | positive |  |
| DBT | | MRPS30-DT | | 0.483 | | | < 0.001 | positive |  |
| DBT | | MYB-AS1 | | 0.557 | | | < 0.001 | positive |  |
| DBT | | NADK2-AS1 | | 0.517 | | | < 0.001 | positive |  |
| DBT | | NBAT1 | | 0.449 | | | < 0.001 | positive |  |
| DBT | | NNT-AS1 | | 0.630 | | | < 0.001 | positive |  |
| DBT | | NORAD | | 0.544 | | | < 0.001 | positive |  |
| DBT | | RP11-15H20.6 | | 0.486 | | | < 0.001 | positive |  |
| DBT | | RP11-541P9.3 | | 0.475 | | | < 0.001 | positive |  |
| DBT | | RP11-655M14.13 | | 0.504 | | | < 0.001 | positive |  |
| DBT | | RP11-97O12.6 | | 0.526 | | | < 0.001 | positive |  |
| DBT | | RRN3P3 | | 0.444 | | | < 0.001 | positive |  |
| DBT | | SATB1-AS1 | | 0.562 | | | < 0.001 | positive |  |
| DBT | | SCOC-AS1 | | 0.477 | | | < 0.001 | positive |  |
| DBT | | SNHG9 | | -0.469 | | | < 0.001 | negative |  |
| DBT | | SP2-AS1 | | 0.672 | | | < 0.001 | positive |  |
| DBT | | SPDYE10P | | 0.504 | | | < 0.001 | positive |  |
| DBT | | SRD5A3-AS1 | | 0.527 | | | < 0.001 | positive |  |
| DBT | | STAG3L3 | | 0.430 | | | < 0.001 | positive |  |
| DBT | | TH2LCRR | | 0.486 | | | < 0.001 | positive |  |
| DBT | | TTC3-AS1 | | 0.594 | | | < 0.001 | positive |  |
| DBT | | VAC14-AS1 | | 0.516 | | | < 0.001 | positive |  |
| DBT | | Z99572.1 | | 0.471 | | | < 0.001 | positive |  |
| DBT | | ZBED3-AS1 | | 0.697 | | | < 0.001 | positive |  |
| DBT | | ZNF137P | | 0.549 | | | < 0.001 | positive |  |
| DBT | | ZNF433-AS1 | | 0.499 | | | < 0.001 | positive |  |
| DBT | | ZNF561-AS1 | | 0.485 | | | < 0.001 | positive |  |
| DBT | | ZNRF2P2 | | 0.438 | | | < 0.001 | positive |  |
| DLAT | | AC005034.6 | | 0.580 | | | < 0.001 | positive |  |
| DLAT | | AC005674.2 | | -0.493 | | | < 0.001 | negative |  |
| DLAT | | AC007298.2 | | -0.516 | | | < 0.001 | negative |  |
| DLAT | | AC010247.2 | | -0.549 | | | < 0.001 | negative |  |
| DLAT | | AC018647.2 | | 0.463 | | | < 0.001 | positive |  |
| DLAT | | AC021224.1 | | 0.564 | | | < 0.001 | positive |  |
| DLAT | | AC022306.2 | | 0.452 | | | < 0.001 | positive |  |
| DLAT | | AC024896.1 | | 0.707 | | | < 0.001 | positive |  |
| DLAT | | AC048351.2 | | 0.441 | | | < 0.001 | positive |  |
| DLAT | | AC087273.2 | | -0.449 | | | < 0.001 | negative |  |
| DLAT | | AC087501.4 | | 0.487 | | | < 0.001 | positive |  |
| DLAT | | AC091563.1 | | 0.569 | | | < 0.001 | positive |  |
| DLAT | | AC096733.3 | | -0.458 | | | < 0.001 | negative |  |
| DLAT | | AC104113.1 | | 0.436 | | | < 0.001 | positive |  |
| DLAT | | AC125603.1 | | 0.528 | | | < 0.001 | positive |  |
| DLAT | | AC133961.1 | | -0.448 | | | < 0.001 | negative |  |
| DLAT | | AL139246.5 | | -0.435 | | | < 0.001 | negative |  |
| DLAT | | AL162311.1 | | 0.518 | | | < 0.001 | positive |  |
| DLAT | | AL390728.6 | | 0.448 | | | < 0.001 | positive |  |
| DLAT | | BX470102.1 | | -0.587 | | | < 0.001 | negative |  |
| DLAT | | CLEC4O | | 0.451 | | | < 0.001 | positive |  |
| DLAT | | CPNE8-AS1 | | -0.480 | | | < 0.001 | negative |  |
| DLAT | | GARS-DT | | -0.466 | | | < 0.001 | negative |  |
| DLAT | | ITGB2-AS1 | | -0.503 | | | < 0.001 | negative |  |
| DLAT | | KB-1674E1.2 | | 0.465 | | | < 0.001 | positive |  |
| DLAT | | LINC00689 | | 0.448 | | | < 0.001 | positive |  |
| DLAT | | LINC00888 | | 0.444 | | | < 0.001 | positive |  |
| DLAT | | LINC01353 | | -0.459 | | | < 0.001 | negative |  |
| DLAT | | LINC01547 | | -0.561 | | | < 0.001 | negative |  |
| DLAT | | LINC01588 | | -0.461 | | | < 0.001 | negative |  |
| DLAT | | NNT-AS1 | | 0.486 | | | < 0.001 | positive |  |
| DLAT | | RP11-541P9.3 | | 0.453 | | | < 0.001 | positive |  |
| DLAT | | RP11-58E21.3 | | -0.467 | | | < 0.001 | negative |  |
| DLAT | | RP11-61L19.3 | | 0.455 | | | < 0.001 | positive |  |
| DLAT | | SCARNA9 | | 0.444 | | | < 0.001 | positive |  |
| DLAT | | SH3BP5-AS1 | | -0.478 | | | < 0.001 | negative |  |
| DLD | | AC004865.2 | | -0.458 | | | < 0.001 | negative |  |
| DLD | | AC007292.2 | | -0.453 | | | < 0.001 | negative |  |
| DLD | | AC021224.1 | | 0.529 | | | < 0.001 | positive |  |
| DLD | | AC024896.1 | | 0.434 | | | < 0.001 | positive |  |
| DLD | | AC245884.8 | | -0.430 | | | < 0.001 | negative |  |
| DLD | | AL844908.2 | | -0.441 | | | < 0.001 | negative |  |
| DLD | | MZF1-AS1 | | -0.448 | | | < 0.001 | negative |  |
| DLST | | AC004112.1 | | -0.549 | | | < 0.001 | negative |  |
| DLST | | AC007161.1 | | -0.501 | | | < 0.001 | negative |  |
| DLST | | AC008074.2 | | -0.479 | | | < 0.001 | negative |  |
| DLST | | AC009554.1 | | -0.563 | | | < 0.001 | negative |  |
| DLST | | AC011978.2 | | -0.561 | | | < 0.001 | negative |  |
| DLST | | AC022960.1 | | -0.463 | | | < 0.001 | negative |  |
| DLST | | AC022960.2 | | -0.570 | | | < 0.001 | negative |  |
| DLST | | AC024475.1 | | -0.576 | | | < 0.001 | negative |  |
| DLST | | AC040904.1 | | -0.464 | | | < 0.001 | negative |  |
| DLST | | AC063943.2 | | -0.465 | | | < 0.001 | negative |  |
| DLST | | AC073534.2 | | -0.473 | | | < 0.001 | negative |  |
| DLST | | AC073569.1 | | -0.560 | | | < 0.001 | negative |  |
| DLST | | AC073934.1 | | -0.606 | | | < 0.001 | negative |  |
| DLST | | AC079915.1 | | -0.506 | | | < 0.001 | negative |  |
| DLST | | AC090651.1 | | -0.471 | | | < 0.001 | negative |  |
| DLST | | AC092436.3 | | -0.472 | | | < 0.001 | negative |  |
| DLST | | AC092794.2 | | -0.453 | | | < 0.001 | negative |  |
| DLST | | AC093752.2 | | -0.452 | | | < 0.001 | negative |  |
| DLST | | AC106028.2 | | -0.476 | | | < 0.001 | negative |  |
| DLST | | AC108102.1 | | -0.505 | | | < 0.001 | negative |  |
| DLST | | AC114980.1 | | -0.432 | | | < 0.001 | negative |  |
| DLST | | AC138028.4 | | 0.477 | | | < 0.001 | positive |  |
| DLST | | AC139792.1 | | -0.454 | | | < 0.001 | negative |  |
| DLST | | AC243960.1 | | 0.434 | | | < 0.001 | positive |  |
| DLST | | AL024474.2 | | -0.488 | | | < 0.001 | negative |  |
| DLST | | AL133243.2 | | -0.471 | | | < 0.001 | negative |  |
| DLST | | AL138955.1 | | -0.506 | | | < 0.001 | negative |  |
| DLST | | AL139120.1 | | -0.447 | | | < 0.001 | negative |  |
| DLST | | AL158163.2 | | -0.435 | | | < 0.001 | negative |  |
| DLST | | AL445933.2 | | -0.484 | | | < 0.001 | negative |  |
| DLST | | AL731568.1 | | -0.436 | | | < 0.001 | negative |  |
| DLST | | AP003168.2 | | -0.483 | | | < 0.001 | negative |  |
| DLST | | AP005482.4 | | -0.517 | | | < 0.001 | negative |  |
| DLST | | BRWD1-IT1 | | -0.608 | | | < 0.001 | negative |  |
| DLST | | DENND6A-AS1 | | -0.520 | | | < 0.001 | negative |  |
| DLST | | GSTCD-AS1 | | -0.494 | | | < 0.001 | negative |  |
| DLST | | HMBOX1-IT1 | | -0.563 | | | < 0.001 | negative |  |
| DLST | | OPA1-AS1 | | -0.439 | | | < 0.001 | negative |  |
| DLST | | PDXDC2P | | -0.499 | | | < 0.001 | negative |  |
| DLST | | RPS27P25 | | -0.538 | | | < 0.001 | negative |  |
| DLST | | SCARNA9 | | -0.432 | | | < 0.001 | negative |  |
| DLST | | Z98257.1 | | -0.486 | | | < 0.001 | negative |  |
| DLST | | ZNF451-AS1 | | -0.549 | | | < 0.001 | negative |  |
| FDX1 | | AC002456.1 | | 0.565 | | | < 0.001 | positive |  |
| FDX1 | | AC005920.2 | | 0.567 | | | < 0.001 | positive |  |
| FDX1 | | AC007161.1 | | 0.459 | | | < 0.001 | positive |  |
| FDX1 | | AC011978.2 | | 0.525 | | | < 0.001 | positive |  |
| FDX1 | | AC012184.3 | | 0.557 | | | < 0.001 | positive |  |
| FDX1 | | AC024475.1 | | 0.488 | | | < 0.001 | positive |  |
| FDX1 | | AC055733.2 | | 0.506 | | | < 0.001 | positive |  |
| FDX1 | | AC063965.2 | | 0.444 | | | < 0.001 | positive |  |
| FDX1 | | AC073569.1 | | 0.478 | | | < 0.001 | positive |  |
| FDX1 | | AC073569.3 | | 0.439 | | | < 0.001 | positive |  |
| FDX1 | | AC073934.1 | | 0.477 | | | < 0.001 | positive |  |
| FDX1 | | AC079684.2 | | 0.580 | | | < 0.001 | positive |  |
| FDX1 | | AC087501.4 | | 0.547 | | | < 0.001 | positive |  |
| FDX1 | | AC090220.1 | | 0.436 | | | < 0.001 | positive |  |
| FDX1 | | AC090970.1 | | 0.475 | | | < 0.001 | positive |  |
| FDX1 | | AC090971.6 | | 0.441 | | | < 0.001 | positive |  |
| FDX1 | | AC092794.2 | | 0.457 | | | < 0.001 | positive |  |
| FDX1 | | AC108102.1 | | 0.447 | | | < 0.001 | positive |  |
| FDX1 | | AC121320.1 | | 0.435 | | | < 0.001 | positive |  |
| FDX1 | | AC135048.1 | | 0.561 | | | < 0.001 | positive |  |
| FDX1 | | AC145207.5 | | 0.485 | | | < 0.001 | positive |  |
| FDX1 | | AL022311.1 | | 0.619 | | | < 0.001 | positive |  |
| FDX1 | | AL136115.2 | | 0.457 | | | < 0.001 | positive |  |
| FDX1 | | AL138955.1 | | 0.532 | | | < 0.001 | positive |  |
| FDX1 | | AL162311.1 | | 0.491 | | | < 0.001 | positive |  |
| FDX1 | | AL353135.1 | | 0.431 | | | < 0.001 | positive |  |
| FDX1 | | AL390728.4 | | 0.445 | | | < 0.001 | positive |  |
| FDX1 | | AP001437.1 | | 0.576 | | | < 0.001 | positive |  |
| FDX1 | | AP005482.4 | | 0.460 | | | < 0.001 | positive |  |
| FDX1 | | BRWD1-IT1 | | 0.480 | | | < 0.001 | positive |  |
| FDX1 | | CDC42-IT1 | | 0.521 | | | < 0.001 | positive |  |
| FDX1 | | EXOSC10-AS1 | | 0.539 | | | < 0.001 | positive |  |
| FDX1 | | FP671120.7 | | 0.533 | | | < 0.001 | positive |  |
| FDX1 | | GIHCG | | 0.431 | | | < 0.001 | positive |  |
| FDX1 | | MIR181A1HG | | 0.446 | | | < 0.001 | positive |  |
| FDX1 | | OPA1-AS1 | | 0.481 | | | < 0.001 | positive |  |
| FDX1 | | PDXDC2P | | 0.522 | | | < 0.001 | positive |  |
| FDX1 | | RC3H1-IT1 | | 0.577 | | | < 0.001 | positive |  |
| FDX1 | | RP11-61L19.3 | | 0.555 | | | < 0.001 | positive |  |
| FDX1 | | RPS27P25 | | 0.575 | | | < 0.001 | positive |  |
| FDX1 | | SCARNA9 | | 0.584 | | | < 0.001 | positive |  |
| FDX1 | | SMAD5-AS1 | | 0.432 | | | < 0.001 | positive |  |
| FDX1 | | SNHG29 | | 0.634 | | | < 0.001 | positive |  |
| FDX1 | | ZFAS1 | | 0.593 | | | < 0.001 | positive |  |
| GCSH | | AC005034.6 | | 0.492 | | | < 0.001 | positive |  |
| GCSH | | AC005674.2 | | -0.480 | | | < 0.001 | negative |  |
| GCSH | | AC010247.2 | | -0.691 | | | < 0.001 | negative |  |
| GCSH | | AC022306.2 | | 0.439 | | | < 0.001 | positive |  |
| GCSH | | AC024896.1 | | 0.482 | | | < 0.001 | positive |  |
| GCSH | | AC025048.6 | | -0.435 | | | < 0.001 | negative |  |
| GCSH | | AC040904.1 | | -0.540 | | | < 0.001 | negative |  |
| GCSH | | AC087273.2 | | -0.748 | | | < 0.001 | negative |  |
| GCSH | | AC090152.1 | | 0.469 | | | < 0.001 | positive |  |
| GCSH | | AC093278.2 | | -0.602 | | | < 0.001 | negative |  |
| GCSH | | AC093583.1 | | -0.466 | | | < 0.001 | negative |  |
| GCSH | | AC096733.3 | | -0.448 | | | < 0.001 | negative |  |
| GCSH | | AC098679.2 | | -0.468 | | | < 0.001 | negative |  |
| GCSH | | AC125603.1 | | 0.434 | | | < 0.001 | positive |  |
| GCSH | | AC133919.2 | | -0.513 | | | < 0.001 | negative |  |
| GCSH | | AC138207.7 | | -0.490 | | | < 0.001 | negative |  |
| GCSH | | ADAMTSL4-AS1 | | -0.482 | | | < 0.001 | negative |  |
| GCSH | | AL133227.1 | | -0.494 | | | < 0.001 | negative |  |
| GCSH | | AL137847.1 | | -0.507 | | | < 0.001 | negative |  |
| GCSH | | AL162311.1 | | 0.549 | | | < 0.001 | positive |  |
| GCSH | | AL353743.1 | | 0.463 | | | < 0.001 | positive |  |
| GCSH | | AL359715.1 | | 0.519 | | | < 0.001 | positive |  |
| GCSH | | AL445489.1 | | -0.436 | | | < 0.001 | negative |  |
| GCSH | | AP000919.4 | | -0.464 | | | < 0.001 | negative |  |
| GCSH | | AP003064.2 | | -0.504 | | | < 0.001 | negative |  |
| GCSH | | B4GALT1-AS1 | | -0.600 | | | < 0.001 | negative |  |
| GCSH | | BX470102.1 | | -0.548 | | | < 0.001 | negative |  |
| GCSH | | CFLAR-AS1 | | -0.512 | | | < 0.001 | negative |  |
| GCSH | | CHROMR | | 0.466 | | | < 0.001 | positive |  |
| GCSH | | DPY19L2P3 | | 0.472 | | | < 0.001 | positive |  |
| GCSH | | EMC3-AS1 | | 0.433 | | | < 0.001 | positive |  |
| GCSH | | GARS-DT | | -0.510 | | | < 0.001 | negative |  |
| GCSH | | GIHCG | | 0.455 | | | < 0.001 | positive |  |
| GCSH | | ITGB2-AS1 | | -0.632 | | | < 0.001 | negative |  |
| GCSH | | JARID2-AS1 | | -0.504 | | | < 0.001 | negative |  |
| GCSH | | LINC00888 | | 0.468 | | | < 0.001 | positive |  |
| GCSH | | LINC01353 | | -0.670 | | | < 0.001 | negative |  |
| GCSH | | LINC01547 | | -0.660 | | | < 0.001 | negative |  |
| GCSH | | LINC01588 | | -0.463 | | | < 0.001 | negative |  |
| GCSH | | LINC02175 | | 0.441 | | | < 0.001 | positive |  |
| GCSH | | LINC02757 | | -0.646 | | | < 0.001 | negative |  |
| GCSH | | LYST-AS1 | | -0.533 | | | < 0.001 | negative |  |
| GCSH | | NNT-AS1 | | 0.442 | | | < 0.001 | positive |  |
| GCSH | | PMS2P4 | | 0.435 | | | < 0.001 | positive |  |
| GCSH | | PSMD6-AS2 | | -0.460 | | | < 0.001 | negative |  |
| GCSH | | RP11-327F22.1 | | -0.588 | | | < 0.001 | negative |  |
| GCSH | | RP11-58E21.3 | | -0.478 | | | < 0.001 | negative |  |
| GCSH | | RPS18P9 | | 0.482 | | | < 0.001 | positive |  |
| GCSH | | SATB1-AS1 | | 0.442 | | | < 0.001 | positive |  |
| GCSH | | SLC8A1-AS1 | | -0.531 | | | < 0.001 | negative |  |
| GCSH | | SNHG29 | | 0.542 | | | < 0.001 | positive |  |
| GCSH | | STAG3L2 | | 0.544 | | | < 0.001 | positive |  |
| GCSH | | TRAF3IP2-AS1 | | 0.428 | | | < 0.001 | positive |  |
| GCSH | | ZFAS1 | | 0.465 | | | < 0.001 | positive |  |
| GCSH | | ZNRF2P2 | | 0.451 | | | < 0.001 | positive |  |
| GLS | | AC000120.2 | | 0.471 | | | < 0.001 | positive |  |
| GLS | | AC002553.2 | | 0.481 | | | < 0.001 | positive |  |
| GLS | | AC004492.1 | | 0.430 | | | < 0.001 | positive |  |
| GLS | | AC004918.5 | | 0.435 | | | < 0.001 | positive |  |
| GLS | | AC005034.6 | | 0.745 | | | < 0.001 | positive |  |
| GLS | | AC006141.1 | | 0.628 | | | < 0.001 | positive |  |
| GLS | | AC006213.5 | | 0.510 | | | < 0.001 | positive |  |
| GLS | | AC006538.1 | | 0.490 | | | < 0.001 | positive |  |
| GLS | | AC007938.3 | | 0.430 | | | < 0.001 | positive |  |
| GLS | | AC008395.1 | | 0.487 | | | < 0.001 | positive |  |
| GLS | | AC008555.1 | | 0.611 | | | < 0.001 | positive |  |
| GLS | | AC009120.2 | | 0.515 | | | < 0.001 | positive |  |
| GLS | | AC009283.1 | | 0.486 | | | < 0.001 | positive |  |
| GLS | | AC009318.2 | | 0.460 | | | < 0.001 | positive |  |
| GLS | | AC010247.2 | | -0.540 | | | < 0.001 | negative |  |
| GLS | | AC010320.4 | | 0.537 | | | < 0.001 | positive |  |
| GLS | | AC010976.1 | | 0.458 | | | < 0.001 | positive |  |
| GLS | | AC011346.1 | | 0.495 | | | < 0.001 | positive |  |
| GLS | | AC011447.3 | | 0.618 | | | < 0.001 | positive |  |
| GLS | | AC011477.1 | | 0.665 | | | < 0.001 | positive |  |
| GLS | | AC011477.3 | | 0.481 | | | < 0.001 | positive |  |
| GLS | | AC012063.1 | | 0.481 | | | < 0.001 | positive |  |
| GLS | | AC012184.3 | | 0.497 | | | < 0.001 | positive |  |
| GLS | | AC015849.3 | | 0.539 | | | < 0.001 | positive |  |
| GLS | | AC018878.1 | | 0.482 | | | < 0.001 | positive |  |
| GLS | | AC020915.1 | | 0.468 | | | < 0.001 | positive |  |
| GLS | | AC021086.1 | | 0.517 | | | < 0.001 | positive |  |
| GLS | | AC021237.1 | | 0.540 | | | < 0.001 | positive |  |
| GLS | | AC021546.1 | | 0.481 | | | < 0.001 | positive |  |
| GLS | | AC022001.2 | | 0.439 | | | < 0.001 | positive |  |
| GLS | | AC022272.1 | | 0.589 | | | < 0.001 | positive |  |
| GLS | | AC022306.2 | | 0.741 | | | < 0.001 | positive |  |
| GLS | | AC022558.1 | | 0.613 | | | < 0.001 | positive |  |
| GLS | | AC022558.3 | | 0.645 | | | < 0.001 | positive |  |
| GLS | | AC022726.1 | | 0.447 | | | < 0.001 | positive |  |
| GLS | | AC024896.1 | | 0.729 | | | < 0.001 | positive |  |
| GLS | | AC024940.6 | | 0.496 | | | < 0.001 | positive |  |
| GLS | | AC025430.1 | | 0.606 | | | < 0.001 | positive |  |
| GLS | | AC025682.2 | | 0.449 | | | < 0.001 | positive |  |
| GLS | | AC026356.2 | | 0.522 | | | < 0.001 | positive |  |
| GLS | | AC026979.3 | | 0.452 | | | < 0.001 | positive |  |
| GLS | | AC034102.8 | | 0.530 | | | < 0.001 | positive |  |
| GLS | | AC048351.2 | | 0.487 | | | < 0.001 | positive |  |
| GLS | | AC048382.1 | | 0.638 | | | < 0.001 | positive |  |
| GLS | | AC055764.1 | | 0.478 | | | < 0.001 | positive |  |
| GLS | | AC068025.1 | | 0.610 | | | < 0.001 | positive |  |
| GLS | | AC068792.1 | | 0.475 | | | < 0.001 | positive |  |
| GLS | | AC073127.1 | | 0.463 | | | < 0.001 | positive |  |
| GLS | | AC073534.2 | | 0.600 | | | < 0.001 | positive |  |
| GLS | | AC078785.2 | | 0.546 | | | < 0.001 | positive |  |
| GLS | | AC078795.1 | | 0.609 | | | < 0.001 | positive |  |
| GLS | | AC079915.1 | | 0.457 | | | < 0.001 | positive |  |
| GLS | | AC083798.2 | | 0.573 | | | < 0.001 | positive |  |
| GLS | | AC083906.3 | | 0.444 | | | < 0.001 | positive |  |
| GLS | | AC084824.4 | | 0.685 | | | < 0.001 | positive |  |
| GLS | | AC087273.2 | | -0.565 | | | < 0.001 | negative |  |
| GLS | | AC087501.4 | | 0.459 | | | < 0.001 | positive |  |
| GLS | | AC090152.1 | | 0.549 | | | < 0.001 | positive |  |
| GLS | | AC090425.2 | | 0.450 | | | < 0.001 | positive |  |
| GLS | | AC090425.3 | | 0.590 | | | < 0.001 | positive |  |
| GLS | | AC090912.2 | | 0.430 | | | < 0.001 | positive |  |
| GLS | | AC091117.3 | | 0.449 | | | < 0.001 | positive |  |
| GLS | | AC091563.1 | | 0.638 | | | < 0.001 | positive |  |
| GLS | | AC092574.1 | | 0.701 | | | < 0.001 | positive |  |
| GLS | | AC092611.1 | | 0.475 | | | < 0.001 | positive |  |
| GLS | | AC092718.5 | | 0.442 | | | < 0.001 | positive |  |
| GLS | | AC093227.1 | | 0.581 | | | < 0.001 | positive |  |
| GLS | | AC096586.2 | | 0.725 | | | < 0.001 | positive |  |
| GLS | | AC103923.1 | | 0.523 | | | < 0.001 | positive |  |
| GLS | | AC104113.1 | | 0.537 | | | < 0.001 | positive |  |
| GLS | | AC104984.4 | | 0.433 | | | < 0.001 | positive |  |
| GLS | | AC104984.6 | | 0.436 | | | < 0.001 | positive |  |
| GLS | | AC109361.1 | | 0.645 | | | < 0.001 | positive |  |
| GLS | | AC110769.2 | | 0.520 | | | < 0.001 | positive |  |
| GLS | | AC112512.1 | | 0.467 | | | < 0.001 | positive |  |
| GLS | | AC113414.1 | | 0.444 | | | < 0.001 | positive |  |
| GLS | | AC120114.1 | | 0.484 | | | < 0.001 | positive |  |
| GLS | | AC121320.1 | | 0.468 | | | < 0.001 | positive |  |
| GLS | | AC124045.1 | | 0.475 | | | < 0.001 | positive |  |
| GLS | | AC127024.4 | | 0.431 | | | < 0.001 | positive |  |
| GLS | | AC127024.5 | | 0.460 | | | < 0.001 | positive |  |
| GLS | | AC129510.1 | | 0.484 | | | < 0.001 | positive |  |
| GLS | | AC129510.2 | | 0.524 | | | < 0.001 | positive |  |
| GLS | | AC130324.1 | | 0.441 | | | < 0.001 | positive |  |
| GLS | | AC132219.1 | | 0.444 | | | < 0.001 | positive |  |
| GLS | | AC133919.2 | | -0.448 | | | < 0.001 | negative |  |
| GLS | | AC138207.6 | | 0.434 | | | < 0.001 | positive |  |
| GLS | | AC211433.1 | | 0.616 | | | < 0.001 | positive |  |
| GLS | | AC211486.1 | | 0.569 | | | < 0.001 | positive |  |
| GLS | | AL022157.1 | | 0.509 | | | < 0.001 | positive |  |
| GLS | | AL023806.1 | | 0.490 | | | < 0.001 | positive |  |
| GLS | | AL031716.1 | | 0.431 | | | < 0.001 | positive |  |
| GLS | | AL080317.2 | | 0.580 | | | < 0.001 | positive |  |
| GLS | | AL109761.1 | | 0.439 | | | < 0.001 | positive |  |
| GLS | | AL133268.3 | | 0.587 | | | < 0.001 | positive |  |
| GLS | | AL138963.1 | | 0.524 | | | < 0.001 | positive |  |
| GLS | | AL139246.5 | | -0.518 | | | < 0.001 | negative |  |
| GLS | | AL139353.2 | | 0.560 | | | < 0.001 | positive |  |
| GLS | | AL161756.1 | | 0.572 | | | < 0.001 | positive |  |
| GLS | | AL162311.1 | | 0.490 | | | < 0.001 | positive |  |
| GLS | | AL353743.1 | | 0.553 | | | < 0.001 | positive |  |
| GLS | | AL353804.2 | | 0.446 | | | < 0.001 | positive |  |
| GLS | | AL354696.2 | | 0.668 | | | < 0.001 | positive |  |
| GLS | | AL356599.1 | | 0.436 | | | < 0.001 | positive |  |
| GLS | | AL359715.1 | | 0.718 | | | < 0.001 | positive |  |
| GLS | | AL359715.3 | | 0.639 | | | < 0.001 | positive |  |
| GLS | | AL390728.4 | | 0.443 | | | < 0.001 | positive |  |
| GLS | | AL390728.6 | | 0.568 | | | < 0.001 | positive |  |
| GLS | | AL442071.1 | | 0.455 | | | < 0.001 | positive |  |
| GLS | | AL512413.1 | | 0.450 | | | < 0.001 | positive |  |
| GLS | | AL590652.1 | | 0.665 | | | < 0.001 | positive |  |
| GLS | | AL591167.1 | | 0.560 | | | < 0.001 | positive |  |
| GLS | | AL591848.4 | | 0.480 | | | < 0.001 | positive |  |
| GLS | | AL592071.1 | | -0.428 | | | < 0.001 | negative |  |
| GLS | | AL645608.4 | | 0.494 | | | < 0.001 | positive |  |
| GLS | | ALG13-AS1 | | 0.474 | | | < 0.001 | positive |  |
| GLS | | ANKRD33B-AS1 | | 0.580 | | | < 0.001 | positive |  |
| GLS | | AP001429.1 | | 0.565 | | | < 0.001 | positive |  |
| GLS | | AP002761.4 | | -0.439 | | | < 0.001 | negative |  |
| GLS | | AP002907.1 | | 0.480 | | | < 0.001 | positive |  |
| GLS | | AP005482.4 | | 0.490 | | | < 0.001 | positive |  |
| GLS | | ARPP21-AS1 | | 0.435 | | | < 0.001 | positive |  |
| GLS | | C9orf147 | | 0.431 | | | < 0.001 | positive |  |
| GLS | | CASC15 | | 0.656 | | | < 0.001 | positive |  |
| GLS | | CCT6P1 | | 0.441 | | | < 0.001 | positive |  |
| GLS | | CCT6P3 | | 0.495 | | | < 0.001 | positive |  |
| GLS | | CHROMR | | 0.453 | | | < 0.001 | positive |  |
| GLS | | DNAJC3-DT | | 0.483 | | | < 0.001 | positive |  |
| GLS | | E2F3-IT1 | | 0.489 | | | < 0.001 | positive |  |
| GLS | | EHMT2-AS1 | | 0.451 | | | < 0.001 | positive |  |
| GLS | | FTX | | 0.604 | | | < 0.001 | positive |  |
| GLS | | GUSBP1 | | 0.454 | | | < 0.001 | positive |  |
| GLS | | HMGN3-AS1 | | 0.565 | | | < 0.001 | positive |  |
| GLS | | IPO5P1 | | 0.518 | | | < 0.001 | positive |  |
| GLS | | ITGB2-AS1 | | -0.474 | | | < 0.001 | negative |  |
| GLS | | KB-1674E1.2 | | 0.480 | | | < 0.001 | positive |  |
| GLS | | KIZ-AS1 | | 0.551 | | | < 0.001 | positive |  |
| GLS | | LINC00539 | | 0.506 | | | < 0.001 | positive |  |
| GLS | | LINC00624 | | 0.677 | | | < 0.001 | positive |  |
| GLS | | LINC00630 | | 0.508 | | | < 0.001 | positive |  |
| GLS | | LINC00674 | | 0.494 | | | < 0.001 | positive |  |
| GLS | | LINC00888 | | 0.429 | | | < 0.001 | positive |  |
| GLS | | LINC00900 | | -0.477 | | | < 0.001 | negative |  |
| GLS | | LINC01353 | | -0.582 | | | < 0.001 | negative |  |
| GLS | | LINC01359 | | 0.537 | | | < 0.001 | positive |  |
| GLS | | LINC01801 | | 0.579 | | | < 0.001 | positive |  |
| GLS | | LINC01963 | | 0.445 | | | < 0.001 | positive |  |
| GLS | | LINC02175 | | 0.496 | | | < 0.001 | positive |  |
| GLS | | LRRC37BP1 | | 0.701 | | | < 0.001 | positive |  |
| GLS | | MESTIT1 | | 0.453 | | | < 0.001 | positive |  |
| GLS | | MFSD13B | | 0.597 | | | < 0.001 | positive |  |
| GLS | | MIPEPP3 | | 0.507 | | | < 0.001 | positive |  |
| GLS | | MIR133A1HG | | 0.505 | | | < 0.001 | positive |  |
| GLS | | MRPS30-DT | | 0.441 | | | < 0.001 | positive |  |
| GLS | | MYB-AS1 | | 0.471 | | | < 0.001 | positive |  |
| GLS | | NADK2-AS1 | | 0.559 | | | < 0.001 | positive |  |
| GLS | | NAPSB | | -0.475 | | | < 0.001 | negative |  |
| GLS | | NBAT1 | | 0.624 | | | < 0.001 | positive |  |
| GLS | | NBR2 | | 0.538 | | | < 0.001 | positive |  |
| GLS | | NNT-AS1 | | 0.739 | | | < 0.001 | positive |  |
| GLS | | NORAD | | 0.518 | | | < 0.001 | positive |  |
| GLS | | OCIAD1-AS1 | | 0.595 | | | < 0.001 | positive |  |
| GLS | | RP11-15H20.6 | | 0.474 | | | < 0.001 | positive |  |
| GLS | | RP11-524C21.2 | | 0.488 | | | < 0.001 | positive |  |
| GLS | | RP11-541P9.3 | | 0.596 | | | < 0.001 | positive |  |
| GLS | | RP11-61L19.3 | | 0.518 | | | < 0.001 | positive |  |
| GLS | | RP11-655M14.13 | | 0.479 | | | < 0.001 | positive |  |
| GLS | | RP11-96G10.1 | | 0.469 | | | < 0.001 | positive |  |
| GLS | | RP11-97O12.6 | | 0.460 | | | < 0.001 | positive |  |
| GLS | | RRN3P3 | | 0.456 | | | < 0.001 | positive |  |
| GLS | | RSF1-IT1 | | 0.443 | | | < 0.001 | positive |  |
| GLS | | SATB1-AS1 | | 0.596 | | | < 0.001 | positive |  |
| GLS | | SCARNA9 | | 0.558 | | | < 0.001 | positive |  |
| GLS | | SCOC-AS1 | | 0.535 | | | < 0.001 | positive |  |
| GLS | | SP2-AS1 | | 0.530 | | | < 0.001 | positive |  |
| GLS | | SPDYE10P | | 0.461 | | | < 0.001 | positive |  |
| GLS | | SRD5A3-AS1 | | 0.463 | | | < 0.001 | positive |  |
| GLS | | STAG3L1 | | 0.438 | | | < 0.001 | positive |  |
| GLS | | STAG3L2 | | 0.530 | | | < 0.001 | positive |  |
| GLS | | STAG3L3 | | 0.545 | | | < 0.001 | positive |  |
| GLS | | STARD4-AS1 | | 0.529 | | | < 0.001 | positive |  |
| GLS | | SUZ12P1 | | 0.484 | | | < 0.001 | positive |  |
| GLS | | SYNGAP1-AS1 | | 0.434 | | | < 0.001 | positive |  |
| GLS | | THAP9-AS1 | | 0.524 | | | < 0.001 | positive |  |
| GLS | | TRAF3IP2-AS1 | | 0.531 | | | < 0.001 | positive |  |
| GLS | | TTC28-AS1 | | 0.467 | | | < 0.001 | positive |  |
| GLS | | TTC3-AS1 | | 0.547 | | | < 0.001 | positive |  |
| GLS | | ZBED3-AS1 | | 0.635 | | | < 0.001 | positive |  |
| GLS | | ZNF433-AS1 | | 0.531 | | | < 0.001 | positive |  |
| GLS | | ZNF561-AS1 | | 0.459 | | | < 0.001 | positive |  |
| LIAS | | AC000120.2 | | 0.452 | | | < 0.001 | positive |  |
| LIAS | | AC004918.5 | | 0.483 | | | < 0.001 | positive |  |
| LIAS | | AC005034.6 | | 0.515 | | | < 0.001 | positive |  |
| LIAS | | AC006116.10 | | 0.446 | | | < 0.001 | positive |  |
| LIAS | | AC006116.7 | | 0.466 | | | < 0.001 | positive |  |
| LIAS | | AC006141.1 | | 0.487 | | | < 0.001 | positive |  |
| LIAS | | AC006213.5 | | 0.664 | | | < 0.001 | positive |  |
| LIAS | | AC007922.3 | | 0.445 | | | < 0.001 | positive |  |
| LIAS | | AC007996.1 | | 0.622 | | | < 0.001 | positive |  |
| LIAS | | AC008395.1 | | 0.454 | | | < 0.001 | positive |  |
| LIAS | | AC008555.1 | | 0.460 | | | < 0.001 | positive |  |
| LIAS | | AC008770.3 | | 0.469 | | | < 0.001 | positive |  |
| LIAS | | AC009095.1 | | 0.456 | | | < 0.001 | positive |  |
| LIAS | | AC009118.3 | | 0.449 | | | < 0.001 | positive |  |
| LIAS | | AC009120.2 | | 0.537 | | | < 0.001 | positive |  |
| LIAS | | AC009120.3 | | 0.460 | | | < 0.001 | positive |  |
| LIAS | | AC009120.5 | | 0.541 | | | < 0.001 | positive |  |
| LIAS | | AC009283.1 | | 0.479 | | | < 0.001 | positive |  |
| LIAS | | AC010247.2 | | -0.470 | | | < 0.001 | negative |  |
| LIAS | | AC010260.1 | | 0.441 | | | < 0.001 | positive |  |
| LIAS | | AC010976.1 | | 0.534 | | | < 0.001 | positive |  |
| LIAS | | AC011005.4 | | 0.448 | | | < 0.001 | positive |  |
| LIAS | | AC011447.3 | | 0.459 | | | < 0.001 | positive |  |
| LIAS | | AC011465.1 | | 0.454 | | | < 0.001 | positive |  |
| LIAS | | AC011477.1 | | 0.518 | | | < 0.001 | positive |  |
| LIAS | | AC011477.3 | | 0.560 | | | < 0.001 | positive |  |
| LIAS | | AC011509.2 | | 0.486 | | | < 0.001 | positive |  |
| LIAS | | AC012063.1 | | 0.679 | | | < 0.001 | positive |  |
| LIAS | | AC015849.3 | | 0.671 | | | < 0.001 | positive |  |
| LIAS | | AC015849.4 | | 0.630 | | | < 0.001 | positive |  |
| LIAS | | AC018645.3 | | 0.474 | | | < 0.001 | positive |  |
| LIAS | | AC018647.2 | | 0.516 | | | < 0.001 | positive |  |
| LIAS | | AC021086.1 | | 0.502 | | | < 0.001 | positive |  |
| LIAS | | AC021224.1 | | 0.502 | | | < 0.001 | positive |  |
| LIAS | | AC021491.1 | | 0.449 | | | < 0.001 | positive |  |
| LIAS | | AC021546.1 | | 0.439 | | | < 0.001 | positive |  |
| LIAS | | AC022272.1 | | 0.504 | | | < 0.001 | positive |  |
| LIAS | | AC022306.2 | | 0.674 | | | < 0.001 | positive |  |
| LIAS | | AC022558.1 | | 0.466 | | | < 0.001 | positive |  |
| LIAS | | AC022558.3 | | 0.553 | | | < 0.001 | positive |  |
| LIAS | | AC024896.1 | | 0.450 | | | < 0.001 | positive |  |
| LIAS | | AC025682.2 | | 0.507 | | | < 0.001 | positive |  |
| LIAS | | AC026356.2 | | 0.603 | | | < 0.001 | positive |  |
| LIAS | | AC027031.2 | | 0.454 | | | < 0.001 | positive |  |
| LIAS | | AC046158.2 | | 0.574 | | | < 0.001 | positive |  |
| LIAS | | AC048351.2 | | 0.537 | | | < 0.001 | positive |  |
| LIAS | | AC048382.1 | | 0.470 | | | < 0.001 | positive |  |
| LIAS | | AC055855.2 | | 0.580 | | | < 0.001 | positive |  |
| LIAS | | AC068025.1 | | 0.742 | | | < 0.001 | positive |  |
| LIAS | | AC073655.2 | | 0.513 | | | < 0.001 | positive |  |
| LIAS | | AC078785.2 | | 0.463 | | | < 0.001 | positive |  |
| LIAS | | AC084824.4 | | 0.459 | | | < 0.001 | positive |  |
| LIAS | | AC087273.2 | | -0.490 | | | < 0.001 | negative |  |
| LIAS | | AC090425.3 | | 0.563 | | | < 0.001 | positive |  |
| LIAS | | AC092574.1 | | 0.523 | | | < 0.001 | positive |  |
| LIAS | | AC092718.1 | | 0.435 | | | < 0.001 | positive |  |
| LIAS | | AC093227.1 | | 0.501 | | | < 0.001 | positive |  |
| LIAS | | AC096586.2 | | 0.497 | | | < 0.001 | positive |  |
| LIAS | | AC098848.1 | | 0.466 | | | < 0.001 | positive |  |
| LIAS | | AC103923.1 | | 0.429 | | | < 0.001 | positive |  |
| LIAS | | AC107214.1 | | 0.485 | | | < 0.001 | positive |  |
| LIAS | | AC107308.1 | | 0.446 | | | < 0.001 | positive |  |
| LIAS | | AC110792.3 | | 0.480 | | | < 0.001 | positive |  |
| LIAS | | AC120114.1 | | 0.451 | | | < 0.001 | positive |  |
| LIAS | | AC127024.5 | | 0.483 | | | < 0.001 | positive |  |
| LIAS | | AC129510.1 | | 0.503 | | | < 0.001 | positive |  |
| LIAS | | AC129510.2 | | 0.459 | | | < 0.001 | positive |  |
| LIAS | | AC130324.1 | | 0.495 | | | < 0.001 | positive |  |
| LIAS | | AC211486.1 | | 0.584 | | | < 0.001 | positive |  |
| LIAS | | AC244093.4 | | 0.502 | | | < 0.001 | positive |  |
| LIAS | | AL031716.1 | | 0.439 | | | < 0.001 | positive |  |
| LIAS | | AL049712.1 | | 0.487 | | | < 0.001 | positive |  |
| LIAS | | AL080317.2 | | 0.565 | | | < 0.001 | positive |  |
| LIAS | | AL132780.2 | | 0.484 | | | < 0.001 | positive |  |
| LIAS | | AL133268.3 | | 0.458 | | | < 0.001 | positive |  |
| LIAS | | AL137186.1 | | 0.464 | | | < 0.001 | positive |  |
| LIAS | | AL138963.1 | | 0.521 | | | < 0.001 | positive |  |
| LIAS | | AL139353.2 | | 0.668 | | | < 0.001 | positive |  |
| LIAS | | AL157400.4 | | 0.563 | | | < 0.001 | positive |  |
| LIAS | | AL158163.2 | | 0.452 | | | < 0.001 | positive |  |
| LIAS | | AL161756.1 | | 0.706 | | | < 0.001 | positive |  |
| LIAS | | AL162727.2 | | 0.515 | | | < 0.001 | positive |  |
| LIAS | | AL353743.1 | | 0.565 | | | < 0.001 | positive |  |
| LIAS | | AL354696.2 | | 0.584 | | | < 0.001 | positive |  |
| LIAS | | AL359715.1 | | 0.489 | | | < 0.001 | positive |  |
| LIAS | | AL359715.3 | | 0.518 | | | < 0.001 | positive |  |
| LIAS | | AL391834.1 | | 0.490 | | | < 0.001 | positive |  |
| LIAS | | AL512413.1 | | 0.537 | | | < 0.001 | positive |  |
| LIAS | | AL590428.1 | | 0.623 | | | < 0.001 | positive |  |
| LIAS | | AL590652.1 | | 0.647 | | | < 0.001 | positive |  |
| LIAS | | AL591167.1 | | 0.446 | | | < 0.001 | positive |  |
| LIAS | | AP000695.1 | | 0.502 | | | < 0.001 | positive |  |
| LIAS | | AP000695.2 | | 0.497 | | | < 0.001 | positive |  |
| LIAS | | AP001107.9 | | 0.448 | | | < 0.001 | positive |  |
| LIAS | | CCT6P1 | | 0.633 | | | < 0.001 | positive |  |
| LIAS | | CHROMR | | 0.433 | | | < 0.001 | positive |  |
| LIAS | | CT66 | | 0.454 | | | < 0.001 | positive |  |
| LIAS | | DNAJC3-DT | | 0.588 | | | < 0.001 | positive |  |
| LIAS | | DOC2GP | | 0.541 | | | < 0.001 | positive |  |
| LIAS | | EHMT2-AS1 | | 0.525 | | | < 0.001 | positive |  |
| LIAS | | FAM215A | | 0.601 | | | < 0.001 | positive |  |
| LIAS | | FAM30A | | 0.449 | | | < 0.001 | positive |  |
| LIAS | | GUSBP5 | | 0.637 | | | < 0.001 | positive |  |
| LIAS | | HLA-F-AS1 | | 0.450 | | | < 0.001 | positive |  |
| LIAS | | HMGA2-AS1 | | 0.559 | | | < 0.001 | positive |  |
| LIAS | | KIZ-AS1 | | 0.466 | | | < 0.001 | positive |  |
| LIAS | | LINC00299 | | 0.481 | | | < 0.001 | positive |  |
| LIAS | | LINC00624 | | 0.583 | | | < 0.001 | positive |  |
| LIAS | | LINC00630 | | 0.436 | | | < 0.001 | positive |  |
| LIAS | | LINC01088 | | 0.578 | | | < 0.001 | positive |  |
| LIAS | | LINC01359 | | 0.557 | | | < 0.001 | positive |  |
| LIAS | | LINC02626 | | 0.489 | | | < 0.001 | positive |  |
| LIAS | | LRRC37A6P | | 0.429 | | | < 0.001 | positive |  |
| LIAS | | LRRC37BP1 | | 0.554 | | | < 0.001 | positive |  |
| LIAS | | MFSD13B | | 0.504 | | | < 0.001 | positive |  |
| LIAS | | MYB-AS1 | | 0.470 | | | < 0.001 | positive |  |
| LIAS | | MYLK-AS2 | | 0.466 | | | < 0.001 | positive |  |
| LIAS | | NBPF25P | | 0.447 | | | < 0.001 | positive |  |
| LIAS | | NNT-AS1 | | 0.626 | | | < 0.001 | positive |  |
| LIAS | | NORAD | | 0.465 | | | < 0.001 | positive |  |
| LIAS | | OCIAD1-AS1 | | 0.511 | | | < 0.001 | positive |  |
| LIAS | | PMS2CL | | 0.448 | | | < 0.001 | positive |  |
| LIAS | | PMS2P4 | | 0.440 | | | < 0.001 | positive |  |
| LIAS | | RP11-327F22.1 | | -0.470 | | | < 0.001 | negative |  |
| LIAS | | RP11-655M14.13 | | 0.599 | | | < 0.001 | positive |  |
| LIAS | | RP11-97O12.6 | | 0.464 | | | < 0.001 | positive |  |
| LIAS | | RPS10P7 | | 0.467 | | | < 0.001 | positive |  |
| LIAS | | RPSAP69 | | 0.477 | | | < 0.001 | positive |  |
| LIAS | | SATB1-AS1 | | 0.544 | | | < 0.001 | positive |  |
| LIAS | | SCOC-AS1 | | 0.499 | | | < 0.001 | positive |  |
| LIAS | | SDHAP3 | | 0.433 | | | < 0.001 | positive |  |
| LIAS | | SH3TC2-DT | | 0.436 | | | < 0.001 | positive |  |
| LIAS | | SP2-AS1 | | 0.529 | | | < 0.001 | positive |  |
| LIAS | | SPDYE10P | | 0.606 | | | < 0.001 | positive |  |
| LIAS | | SRD5A3-AS1 | | 0.497 | | | < 0.001 | positive |  |
| LIAS | | STAG3L3 | | 0.448 | | | < 0.001 | positive |  |
| LIAS | | TH2LCRR | | 0.473 | | | < 0.001 | positive |  |
| LIAS | | TPM3P9 | | 0.481 | | | < 0.001 | positive |  |
| LIAS | | TTC28-AS1 | | 0.518 | | | < 0.001 | positive |  |
| LIAS | | Z99572.1 | | 0.543 | | | < 0.001 | positive |  |
| LIAS | | ZBED3-AS1 | | 0.519 | | | < 0.001 | positive |  |
| LIAS | | ZNF137P | | 0.460 | | | < 0.001 | positive |  |
| LIAS | | ZNF451-AS1 | | 0.429 | | | < 0.001 | positive |  |
| LIAS | | ZNF561-AS1 | | 0.441 | | | < 0.001 | positive |  |
| LIPT1 | | AC004112.1 | | 0.524 | | | < 0.001 | positive |  |
| LIPT1 | | AC004951.4 | | 0.490 | | | < 0.001 | positive |  |
| LIPT1 | | AC005476.2 | | -0.438 | | | < 0.001 | negative |  |
| LIPT1 | | AC005696.1 | | 0.489 | | | < 0.001 | positive |  |
| LIPT1 | | AC006504.2 | | 0.473 | | | < 0.001 | positive |  |
| LIPT1 | | AC007038.1 | | 0.464 | | | < 0.001 | positive |  |
| LIPT1 | | AC007922.3 | | 0.615 | | | < 0.001 | positive |  |
| LIPT1 | | AC008543.5 | | 0.547 | | | < 0.001 | positive |  |
| LIPT1 | | AC008738.2 | | 0.466 | | | < 0.001 | positive |  |
| LIPT1 | | AC008770.3 | | 0.509 | | | < 0.001 | positive |  |
| LIPT1 | | AC009120.5 | | 0.485 | | | < 0.001 | positive |  |
| LIPT1 | | AC010320.4 | | 0.509 | | | < 0.001 | positive |  |
| LIPT1 | | AC011120.1 | | 0.430 | | | < 0.001 | positive |  |
| LIPT1 | | AC011447.3 | | 0.474 | | | < 0.001 | positive |  |
| LIPT1 | | AC011477.1 | | 0.555 | | | < 0.001 | positive |  |
| LIPT1 | | AC020915.2 | | 0.503 | | | < 0.001 | positive |  |
| LIPT1 | | AC022150.2 | | 0.437 | | | < 0.001 | positive |  |
| LIPT1 | | AC022154.1 | | 0.494 | | | < 0.001 | positive |  |
| LIPT1 | | AC022272.1 | | 0.482 | | | < 0.001 | positive |  |
| LIPT1 | | AC022415.2 | | 0.612 | | | < 0.001 | positive |  |
| LIPT1 | | AC026979.3 | | 0.442 | | | < 0.001 | positive |  |
| LIPT1 | | AC046158.2 | | 0.445 | | | < 0.001 | positive |  |
| LIPT1 | | AC078795.1 | | 0.448 | | | < 0.001 | positive |  |
| LIPT1 | | AC079322.1 | | 0.496 | | | < 0.001 | positive |  |
| LIPT1 | | AC084357.2 | | 0.466 | | | < 0.001 | positive |  |
| LIPT1 | | AC090425.3 | | 0.502 | | | < 0.001 | positive |  |
| LIPT1 | | AC093227.1 | | 0.530 | | | < 0.001 | positive |  |
| LIPT1 | | AC097376.3 | | 0.565 | | | < 0.001 | positive |  |
| LIPT1 | | AC103987.3 | | 0.555 | | | < 0.001 | positive |  |
| LIPT1 | | AC104113.1 | | 0.517 | | | < 0.001 | positive |  |
| LIPT1 | | AC104964.1 | | 0.539 | | | < 0.001 | positive |  |
| LIPT1 | | AC104964.3 | | 0.444 | | | < 0.001 | positive |  |
| LIPT1 | | AC104964.4 | | 0.440 | | | < 0.001 | positive |  |
| LIPT1 | | AC106028.2 | | 0.465 | | | < 0.001 | positive |  |
| LIPT1 | | AC106028.3 | | 0.441 | | | < 0.001 | positive |  |
| LIPT1 | | AC109361.1 | | 0.468 | | | < 0.001 | positive |  |
| LIPT1 | | AC130324.1 | | 0.502 | | | < 0.001 | positive |  |
| LIPT1 | | AC130352.1 | | 0.502 | | | < 0.001 | positive |  |
| LIPT1 | | AC245884.8 | | 0.431 | | | < 0.001 | positive |  |
| LIPT1 | | AL035458.2 | | 0.455 | | | < 0.001 | positive |  |
| LIPT1 | | AL118558.4 | | 0.441 | | | < 0.001 | positive |  |
| LIPT1 | | AL135999.2 | | 0.498 | | | < 0.001 | positive |  |
| LIPT1 | | AL139353.2 | | 0.567 | | | < 0.001 | positive |  |
| LIPT1 | | AL161756.1 | | 0.540 | | | < 0.001 | positive |  |
| LIPT1 | | AL359715.3 | | 0.525 | | | < 0.001 | positive |  |
| LIPT1 | | AL583856.2 | | 0.536 | | | < 0.001 | positive |  |
| LIPT1 | | AP000790.1 | | 0.570 | | | < 0.001 | positive |  |
| LIPT1 | | CHROMR | | 0.494 | | | < 0.001 | positive |  |
| LIPT1 | | CTD-2521M24.9 | | 0.506 | | | < 0.001 | positive |  |
| LIPT1 | | DNAJC3-DT | | 0.633 | | | < 0.001 | positive |  |
| LIPT1 | | INTS4P2 | | 0.499 | | | < 0.001 | positive |  |
| LIPT1 | | MSH2-OT1 | | 0.445 | | | < 0.001 | positive |  |
| LIPT1 | | RP11-327F22.1 | | -0.446 | | | < 0.001 | negative |  |
| LIPT1 | | RPS10P7 | | 0.466 | | | < 0.001 | positive |  |
| LIPT1 | | RPS18P9 | | 0.542 | | | < 0.001 | positive |  |
| LIPT1 | | SCOC-AS1 | | 0.501 | | | < 0.001 | positive |  |
| LIPT1 | | SPDYE10P | | 0.618 | | | < 0.001 | positive |  |
| LIPT1 | | WHAMMP2 | | 0.466 | | | < 0.001 | positive |  |
| LIPT1 | | WHAMMP3 | | 0.448 | | | < 0.001 | positive |  |
| LIPT1 | | Z98257.1 | | 0.516 | | | < 0.001 | positive |  |
| LIPT1 | | ZNF433-AS1 | | 0.571 | | | < 0.001 | positive |  |
| LIPT1 | | ZNF561-AS1 | | 0.555 | | | < 0.001 | positive |  |
| LIPT1 | | ZNF790-AS1 | | 0.441 | | | < 0.001 | positive |  |
| LIPT2 | | AC004918.5 | | 0.469 | | | < 0.001 | positive |  |
| LIPT2 | | AC010247.2 | | -0.437 | | | < 0.001 | negative |  |
| LIPT2 | | AC010320.4 | | 0.488 | | | < 0.001 | positive |  |
| LIPT2 | | AC087392.3 | | 0.471 | | | < 0.001 | positive |  |
| LIPT2 | | AC093627.5 | | 0.433 | | | < 0.001 | positive |  |
| LIPT2 | | AC244502.3 | | 0.436 | | | < 0.001 | positive |  |
| LIPT2 | | AF274858.1 | | 0.445 | | | < 0.001 | positive |  |
| LIPT2 | | AL118558.4 | | 0.458 | | | < 0.001 | positive |  |
| LIPT2 | | CBX3P4 | | 0.443 | | | < 0.001 | positive |  |
| LIPT2 | | CCT6P1 | | 0.469 | | | < 0.001 | positive |  |
| LIPT2 | | CCT6P3 | | 0.465 | | | < 0.001 | positive |  |
| LIPT2 | | DPY19L2P3 | | 0.470 | | | < 0.001 | positive |  |
| LIPT2 | | IPO5P1 | | 0.485 | | | < 0.001 | positive |  |
| LIPT2 | | LINC00888 | | 0.450 | | | < 0.001 | positive |  |
| LIPT2 | | RP11-282O18.3 | | 0.435 | | | < 0.001 | positive |  |
| LIPT2 | | SPART-AS1 | | 0.512 | | | < 0.001 | positive |  |
| LIPT2 | | STAG3L1 | | 0.443 | | | < 0.001 | positive |  |
| LIPT2 | | STAG3L2 | | 0.446 | | | < 0.001 | positive |  |
| LIPT2 | | ZNRF2P2 | | 0.484 | | | < 0.001 | positive |  |
| MTF1 | | AC005476.2 | | 0.559 | | | < 0.001 | positive |  |
| MTF1 | | AC007546.1 | | 0.460 | | | < 0.001 | positive |  |
| MTF1 | | AC009716.1 | | 0.519 | | | < 0.001 | positive |  |
| MTF1 | | AC010247.2 | | 0.554 | | | < 0.001 | positive |  |
| MTF1 | | AC012184.3 | | -0.470 | | | < 0.001 | negative |  |
| MTF1 | | AC012645.3 | | 0.471 | | | < 0.001 | positive |  |
| MTF1 | | AC025048.6 | | 0.434 | | | < 0.001 | positive |  |
| MTF1 | | AC040904.1 | | 0.493 | | | < 0.001 | positive |  |
| MTF1 | | AC068792.1 | | -0.459 | | | < 0.001 | negative |  |
| MTF1 | | AC073342.2 | | 0.546 | | | < 0.001 | positive |  |
| MTF1 | | AC087273.2 | | 0.614 | | | < 0.001 | positive |  |
| MTF1 | | AC090152.1 | | -0.574 | | | < 0.001 | negative |  |
| MTF1 | | AC092718.1 | | -0.446 | | | < 0.001 | negative |  |
| MTF1 | | AC093227.1 | | -0.438 | | | < 0.001 | negative |  |
| MTF1 | | AC093278.2 | | 0.584 | | | < 0.001 | positive |  |
| MTF1 | | AC093583.1 | | 0.447 | | | < 0.001 | positive |  |
| MTF1 | | AC098679.2 | | 0.452 | | | < 0.001 | positive |  |
| MTF1 | | AC107204.1 | | -0.483 | | | < 0.001 | negative |  |
| MTF1 | | AC211433.1 | | -0.466 | | | < 0.001 | negative |  |
| MTF1 | | ADAMTSL4-AS1 | | 0.461 | | | < 0.001 | positive |  |
| MTF1 | | AL133268.3 | | -0.438 | | | < 0.001 | negative |  |
| MTF1 | | AL353743.1 | | -0.503 | | | < 0.001 | negative |  |
| MTF1 | | AL355312.2 | | 0.571 | | | < 0.001 | positive |  |
| MTF1 | | AL355353.2 | | -0.517 | | | < 0.001 | negative |  |
| MTF1 | | AL359715.1 | | -0.588 | | | < 0.001 | negative |  |
| MTF1 | | AL445489.1 | | 0.437 | | | < 0.001 | positive |  |
| MTF1 | | AL590652.1 | | -0.461 | | | < 0.001 | negative |  |
| MTF1 | | AP000919.4 | | 0.463 | | | < 0.001 | positive |  |
| MTF1 | | AP002761.4 | | 0.448 | | | < 0.001 | positive |  |
| MTF1 | | AP003064.2 | | 0.440 | | | < 0.001 | positive |  |
| MTF1 | | B4GALT1-AS1 | | 0.549 | | | < 0.001 | positive |  |
| MTF1 | | BX470102.1 | | 0.499 | | | < 0.001 | positive |  |
| MTF1 | | CFLAR-AS1 | | 0.639 | | | < 0.001 | positive |  |
| MTF1 | | CHROMR | | -0.556 | | | < 0.001 | negative |  |
| MTF1 | | CT66 | | -0.428 | | | < 0.001 | negative |  |
| MTF1 | | CXCR2P1 | | 0.460 | | | < 0.001 | positive |  |
| MTF1 | | CYTOR | | -0.442 | | | < 0.001 | negative |  |
| MTF1 | | DENND5B-AS1 | | -0.496 | | | < 0.001 | negative |  |
| MTF1 | | GARS-DT | | 0.465 | | | < 0.001 | positive |  |
| MTF1 | | GIHCG | | -0.448 | | | < 0.001 | negative |  |
| MTF1 | | GUSBP1 | | -0.432 | | | < 0.001 | negative |  |
| MTF1 | | IPO5P1 | | -0.443 | | | < 0.001 | negative |  |
| MTF1 | | LINC00539 | | -0.466 | | | < 0.001 | negative |  |
| MTF1 | | LINC00624 | | -0.450 | | | < 0.001 | negative |  |
| MTF1 | | LINC00888 | | -0.461 | | | < 0.001 | negative |  |
| MTF1 | | LINC01353 | | 0.681 | | | < 0.001 | positive |  |
| MTF1 | | LINC01547 | | 0.684 | | | < 0.001 | positive |  |
| MTF1 | | LINC02757 | | 0.724 | | | < 0.001 | positive |  |
| MTF1 | | LYST-AS1 | | 0.679 | | | < 0.001 | positive |  |
| MTF1 | | MIPEPP3 | | -0.512 | | | < 0.001 | negative |  |
| MTF1 | | NNT-AS1 | | -0.432 | | | < 0.001 | negative |  |
| MTF1 | | PMS2P4 | | -0.466 | | | < 0.001 | negative |  |
| MTF1 | | RP11-15H20.6 | | -0.435 | | | < 0.001 | negative |  |
| MTF1 | | RP11-327F22.1 | | 0.568 | | | < 0.001 | positive |  |
| MTF1 | | RPS18P9 | | -0.461 | | | < 0.001 | negative |  |
| MTF1 | | SATB1-AS1 | | -0.490 | | | < 0.001 | negative |  |
| MTF1 | | SCOC-AS1 | | -0.493 | | | < 0.001 | negative |  |
| MTF1 | | SLC8A1-AS1 | | 0.440 | | | < 0.001 | positive |  |
| MTF1 | | STAG3L2 | | -0.542 | | | < 0.001 | negative |  |
| MTF1 | | STAG3L3 | | -0.514 | | | < 0.001 | negative |  |
| MTF1 | | STARD4-AS1 | | -0.440 | | | < 0.001 | negative |  |
| MTF1 | | TRAF3IP2-AS1 | | -0.484 | | | < 0.001 | negative |  |
| MTF1 | | TTC28-AS1 | | -0.500 | | | < 0.001 | negative |  |
| MTF1 | | ZFAS1 | | -0.498 | | | < 0.001 | negative |  |
| MTF1 | | ZNF561-AS1 | | -0.477 | | | < 0.001 | negative |  |
| MTF1 | | ZNF790-AS1 | | -0.456 | | | < 0.001 | negative |  |
| NFE2L2 | | AC011471.2 | | 0.452 | | | < 0.001 | positive |  |
| NFE2L2 | | AC012568.1 | | 0.464 | | | < 0.001 | positive |  |
| NFE2L2 | | AC018926.3 | | 0.447 | | | < 0.001 | positive |  |
| NFE2L2 | | AC040904.1 | | 0.518 | | | < 0.001 | positive |  |
| NFE2L2 | | AC090152.1 | | -0.490 | | | < 0.001 | negative |  |
| NFE2L2 | | AC093278.2 | | 0.455 | | | < 0.001 | positive |  |
| NFE2L2 | | AC098679.2 | | 0.437 | | | < 0.001 | positive |  |
| NFE2L2 | | AF274858.1 | | -0.444 | | | < 0.001 | negative |  |
| NFE2L2 | | AL390774.2 | | 0.446 | | | < 0.001 | positive |  |
| NFE2L2 | | AL445489.1 | | 0.496 | | | < 0.001 | positive |  |
| NFE2L2 | | AL445933.2 | | 0.533 | | | < 0.001 | positive |  |
| NFE2L2 | | CFLAR-AS1 | | 0.493 | | | < 0.001 | positive |  |
| NFE2L2 | | DHRSX-IT1 | | 0.469 | | | < 0.001 | positive |  |
| NFE2L2 | | LINC00539 | | -0.466 | | | < 0.001 | negative |  |
| NFE2L2 | | LYST-AS1 | | 0.510 | | | < 0.001 | positive |  |
| NFE2L2 | | MIPEPP3 | | -0.495 | | | < 0.001 | negative |  |
| NFE2L2 | | SNHG9 | | -0.440 | | | 0.001 | negative |  |
| NLRP3 | | AC005034.6 | | -0.564 | | | < 0.001 | negative |  |
| NLRP3 | | AC005476.2 | | 0.499 | | | < 0.001 | positive |  |
| NLRP3 | | AC005674.2 | | 0.437 | | | 0.001 | positive |  |
| NLRP3 | | AC006116.7 | | -0.475 | | | < 0.001 | negative |  |
| NLRP3 | | AC006213.5 | | -0.474 | | | < 0.001 | negative |  |
| NLRP3 | | AC006538.1 | | -0.515 | | | < 0.001 | negative |  |
| NLRP3 | | AC008474.1 | | -0.442 | | | 0.001 | negative |  |
| NLRP3 | | AC008738.2 | | -0.442 | | | 0.001 | negative |  |
| NLRP3 | | AC009120.2 | | -0.468 | | | < 0.001 | negative |  |
| NLRP3 | | AC009563.1 | | -0.459 | | | < 0.001 | negative |  |
| NLRP3 | | AC009716.1 | | 0.454 | | | < 0.001 | positive |  |
| NLRP3 | | AC010247.2 | | 0.699 | | | < 0.001 | positive |  |
| NLRP3 | | AC011346.1 | | -0.475 | | | < 0.001 | negative |  |
| NLRP3 | | AC011447.3 | | -0.497 | | | < 0.001 | negative |  |
| NLRP3 | | AC011477.1 | | -0.495 | | | < 0.001 | negative |  |
| NLRP3 | | AC020915.1 | | -0.528 | | | < 0.001 | negative |  |
| NLRP3 | | AC020915.3 | | -0.491 | | | < 0.001 | negative |  |
| NLRP3 | | AC021086.1 | | -0.449 | | | 0.001 | negative |  |
| NLRP3 | | AC022150.2 | | -0.443 | | | 0.001 | negative |  |
| NLRP3 | | AC022306.2 | | -0.440 | | | 0.001 | negative |  |
| NLRP3 | | AC024896.1 | | -0.630 | | | < 0.001 | negative |  |
| NLRP3 | | AC040904.1 | | 0.432 | | | 0.001 | positive |  |
| NLRP3 | | AC048351.2 | | -0.467 | | | < 0.001 | negative |  |
| NLRP3 | | AC048382.1 | | -0.475 | | | < 0.001 | negative |  |
| NLRP3 | | AC068025.1 | | -0.534 | | | < 0.001 | negative |  |
| NLRP3 | | AC073127.1 | | -0.491 | | | < 0.001 | negative |  |
| NLRP3 | | AC073534.2 | | -0.463 | | | < 0.001 | negative |  |
| NLRP3 | | AC078795.1 | | -0.540 | | | < 0.001 | negative |  |
| NLRP3 | | AC087273.2 | | 0.648 | | | < 0.001 | positive |  |
| NLRP3 | | AC090152.1 | | -0.434 | | | 0.001 | negative |  |
| NLRP3 | | AC090425.3 | | -0.475 | | | < 0.001 | negative |  |
| NLRP3 | | AC091563.1 | | -0.471 | | | < 0.001 | negative |  |
| NLRP3 | | AC092574.1 | | -0.444 | | | 0.001 | negative |  |
| NLRP3 | | AC093227.1 | | -0.479 | | | < 0.001 | negative |  |
| NLRP3 | | AC093278.2 | | 0.573 | | | < 0.001 | positive |  |
| NLRP3 | | AC093583.1 | | 0.538 | | | < 0.001 | positive |  |
| NLRP3 | | AC109361.1 | | -0.490 | | | < 0.001 | negative |  |
| NLRP3 | | AC110769.2 | | -0.435 | | | 0.001 | negative |  |
| NLRP3 | | AC120114.1 | | -0.502 | | | < 0.001 | negative |  |
| NLRP3 | | AC129510.1 | | -0.488 | | | < 0.001 | negative |  |
| NLRP3 | | AC129510.2 | | -0.445 | | | 0.001 | negative |  |
| NLRP3 | | AC133919.2 | | 0.520 | | | < 0.001 | positive |  |
| NLRP3 | | AC211433.1 | | -0.469 | | | < 0.001 | negative |  |
| NLRP3 | | AC211486.1 | | -0.491 | | | < 0.001 | negative |  |
| NLRP3 | | ADAMTSL4-AS1 | | 0.631 | | | < 0.001 | positive |  |
| NLRP3 | | AL080317.2 | | -0.472 | | | < 0.001 | negative |  |
| NLRP3 | | AL133268.3 | | -0.563 | | | < 0.001 | negative |  |
| NLRP3 | | AL161756.1 | | -0.476 | | | < 0.001 | negative |  |
| NLRP3 | | AL162311.1 | | -0.440 | | | 0.001 | negative |  |
| NLRP3 | | AL353743.1 | | -0.595 | | | < 0.001 | negative |  |
| NLRP3 | | AL354696.2 | | -0.538 | | | < 0.001 | negative |  |
| NLRP3 | | AL359715.1 | | -0.614 | | | < 0.001 | negative |  |
| NLRP3 | | AL359715.3 | | -0.497 | | | < 0.001 | negative |  |
| NLRP3 | | AL590652.1 | | -0.536 | | | < 0.001 | negative |  |
| NLRP3 | | AL591167.1 | | -0.506 | | | < 0.001 | negative |  |
| NLRP3 | | AL591848.4 | | -0.476 | | | < 0.001 | negative |  |
| NLRP3 | | ANKRD33B-AS1 | | -0.610 | | | < 0.001 | negative |  |
| NLRP3 | | AP002761.4 | | 0.434 | | | 0.001 | positive |  |
| NLRP3 | | AP003064.2 | | 0.491 | | | < 0.001 | positive |  |
| NLRP3 | | B4GALT1-AS1 | | 0.749 | | | < 0.001 | positive |  |
| NLRP3 | | BX470102.1 | | 0.448 | | | 0.001 | positive |  |
| NLRP3 | | C9orf147 | | -0.443 | | | 0.001 | negative |  |
| NLRP3 | | CASC15 | | -0.462 | | | < 0.001 | negative |  |
| NLRP3 | | CCT6P1 | | -0.439 | | | 0.001 | negative |  |
| NLRP3 | | CHROMR | | -0.510 | | | < 0.001 | negative |  |
| NLRP3 | | DIAPH1-AS1 | | 0.432 | | | 0.001 | positive |  |
| NLRP3 | | DNAJC3-DT | | -0.543 | | | < 0.001 | negative |  |
| NLRP3 | | DPY19L2P3 | | -0.530 | | | < 0.001 | negative |  |
| NLRP3 | | EMC3-AS1 | | -0.449 | | | 0.001 | negative |  |
| NLRP3 | | GUSBP5 | | -0.434 | | | 0.001 | negative |  |
| NLRP3 | | IPO5P1 | | -0.516 | | | < 0.001 | negative |  |
| NLRP3 | | ITGB2-AS1 | | 0.469 | | | < 0.001 | positive |  |
| NLRP3 | | KB-1674E1.2 | | -0.445 | | | 0.001 | negative |  |
| NLRP3 | | KIZ-AS1 | | -0.493 | | | < 0.001 | negative |  |
| NLRP3 | | LINC00539 | | -0.473 | | | < 0.001 | negative |  |
| NLRP3 | | LINC00624 | | -0.536 | | | < 0.001 | negative |  |
| NLRP3 | | LINC00630 | | -0.460 | | | < 0.001 | negative |  |
| NLRP3 | | LINC00674 | | -0.569 | | | < 0.001 | negative |  |
| NLRP3 | | LINC00888 | | -0.458 | | | < 0.001 | negative |  |
| NLRP3 | | LINC01353 | | 0.721 | | | < 0.001 | positive |  |
| NLRP3 | | LINC01547 | | 0.525 | | | < 0.001 | positive |  |
| NLRP3 | | LINC01801 | | -0.434 | | | 0.001 | negative |  |
| NLRP3 | | LINC02175 | | -0.577 | | | < 0.001 | negative |  |
| NLRP3 | | LINC02757 | | 0.584 | | | < 0.001 | positive |  |
| NLRP3 | | LRRC37BP1 | | -0.507 | | | < 0.001 | negative |  |
| NLRP3 | | LYST-AS1 | | 0.429 | | | 0.001 | positive |  |
| NLRP3 | | MIPEPP3 | | -0.508 | | | < 0.001 | negative |  |
| NLRP3 | | MRPS30-DT | | -0.478 | | | < 0.001 | negative |  |
| NLRP3 | | MYB-AS1 | | -0.464 | | | < 0.001 | negative |  |
| NLRP3 | | NADK2-AS1 | | -0.604 | | | < 0.001 | negative |  |
| NLRP3 | | NAPSB | | 0.521 | | | < 0.001 | positive |  |
| NLRP3 | | NNT-AS1 | | -0.618 | | | < 0.001 | negative |  |
| NLRP3 | | OCIAD1-AS1 | | -0.447 | | | 0.001 | negative |  |
| NLRP3 | | PMS2CL | | -0.446 | | | 0.001 | negative |  |
| NLRP3 | | PMS2P4 | | -0.472 | | | < 0.001 | negative |  |
| NLRP3 | | PSMB8-AS1 | | 0.438 | | | 0.001 | positive |  |
| NLRP3 | | RP11-15H20.6 | | -0.513 | | | < 0.001 | negative |  |
| NLRP3 | | RP11-327F22.1 | | 0.610 | | | < 0.001 | positive |  |
| NLRP3 | | RP11-541P9.3 | | -0.527 | | | < 0.001 | negative |  |
| NLRP3 | | RRN3P3 | | -0.435 | | | 0.001 | negative |  |
| NLRP3 | | SATB1-AS1 | | -0.488 | | | < 0.001 | negative |  |
| NLRP3 | | SCOC-AS1 | | -0.524 | | | < 0.001 | negative |  |
| NLRP3 | | SPDYE10P | | -0.471 | | | < 0.001 | negative |  |
| NLRP3 | | STAG3L1 | | -0.436 | | | 0.001 | negative |  |
| NLRP3 | | STAG3L2 | | -0.632 | | | < 0.001 | negative |  |
| NLRP3 | | STAG3L3 | | -0.563 | | | < 0.001 | negative |  |
| NLRP3 | | STARD4-AS1 | | -0.538 | | | < 0.001 | negative |  |
| NLRP3 | | TRAF3IP2-AS1 | | -0.504 | | | < 0.001 | negative |  |
| NLRP3 | | TTC28-AS1 | | -0.580 | | | < 0.001 | negative |  |
| NLRP3 | | Z99572.1 | | -0.526 | | | < 0.001 | negative |  |
| NLRP3 | | ZBED3-AS1 | | -0.501 | | | < 0.001 | negative |  |
| NLRP3 | | ZNF137P | | -0.439 | | | 0.001 | negative |  |
| NLRP3 | | ZNF433-AS1 | | -0.507 | | | < 0.001 | negative |  |
| NLRP3 | | ZNF561-AS1 | | -0.506 | | | < 0.001 | negative |  |
| NLRP3 | | ZNF790-AS1 | | -0.481 | | | < 0.001 | negative |  |
| NLRP3 | | ZNRF2P2 | | -0.581 | | | < 0.001 | negative |  |
| PDHA1 | | AC010247.2 | | -0.448 | | | 0.001 | negative |  |
| PDHA1 | | AC015712.2 | | 0.445 | | | 0.001 | positive |  |
| PDHA1 | | AC022960.1 | | -0.497 | | | < 0.001 | negative |  |
| PDHA1 | | AC024475.1 | | -0.475 | | | < 0.001 | negative |  |
| PDHA1 | | AC026904.1 | | 0.462 | | | < 0.001 | positive |  |
| PDHA1 | | AL354920.1 | | 0.457 | | | < 0.001 | positive |  |
| PDHA1 | | AP003168.2 | | -0.457 | | | < 0.001 | negative |  |
| PDHA1 | | BRWD1-IT1 | | -0.461 | | | < 0.001 | negative |  |
| PDHA1 | | HMBOX1-IT1 | | -0.467 | | | < 0.001 | negative |  |
| PDHA1 | | RP11-327F22.1 | | -0.450 | | | < 0.001 | negative |  |
| PDHA1 | | SCAANT1 | | -0.428 | | | 0.001 | negative |  |
| PDHB | | AC104958.2 | | -0.483 | | | < 0.001 | negative |  |
| PDHB | | AC125807.2 | | 0.492 | | | < 0.001 | positive |  |
| PDHB | | AL133215.2 | | -0.451 | | | < 0.001 | negative |  |
| PDHB | | CTD-2245F17.3 | | -0.443 | | | 0.001 | negative |  |
| PDHB | | HLA-F-AS1 | | -0.495 | | | < 0.001 | negative |  |
| PDHB | | LINC00987 | | -0.474 | | | < 0.001 | negative |  |
| PDHB | | LINC01624 | | -0.443 | | | 0.001 | negative |  |
| PDHB | | LMNTD2-AS1 | | -0.468 | | | < 0.001 | negative |  |
| PDHB | | PMS2CL | | -0.482 | | | < 0.001 | negative |  |
| SLC31A1 | | AC004241.1 | | 0.496 | | | < 0.001 | positive |  |
| SLC31A1 | | AC005476.2 | | 0.522 | | | < 0.001 | positive |  |
| SLC31A1 | | AC006116.7 | | -0.451 | | | < 0.001 | negative |  |
| SLC31A1 | | AC009563.1 | | -0.453 | | | < 0.001 | negative |  |
| SLC31A1 | | AC009996.1 | | -0.441 | | | 0.001 | negative |  |
| SLC31A1 | | AC010247.2 | | 0.742 | | | < 0.001 | positive |  |
| SLC31A1 | | AC010320.4 | | -0.507 | | | < 0.001 | negative |  |
| SLC31A1 | | AC011447.3 | | -0.482 | | | < 0.001 | negative |  |
| SLC31A1 | | AC011477.1 | | -0.495 | | | < 0.001 | negative |  |
| SLC31A1 | | AC020915.1 | | -0.513 | | | < 0.001 | negative |  |
| SLC31A1 | | AC020915.2 | | -0.473 | | | < 0.001 | negative |  |
| SLC31A1 | | AC020915.3 | | -0.488 | | | < 0.001 | negative |  |
| SLC31A1 | | AC022007.1 | | -0.453 | | | < 0.001 | negative |  |
| SLC31A1 | | AC022150.2 | | -0.460 | | | < 0.001 | negative |  |
| SLC31A1 | | AC022415.2 | | -0.433 | | | 0.001 | negative |  |
| SLC31A1 | | AC024896.1 | | -0.467 | | | < 0.001 | negative |  |
| SLC31A1 | | AC026356.2 | | -0.445 | | | 0.001 | negative |  |
| SLC31A1 | | AC040904.1 | | 0.457 | | | < 0.001 | positive |  |
| SLC31A1 | | AC073127.1 | | -0.438 | | | 0.001 | negative |  |
| SLC31A1 | | AC073534.2 | | -0.434 | | | 0.001 | negative |  |
| SLC31A1 | | AC078785.2 | | -0.483 | | | < 0.001 | negative |  |
| SLC31A1 | | AC078795.1 | | -0.485 | | | < 0.001 | negative |  |
| SLC31A1 | | AC087273.2 | | 0.726 | | | < 0.001 | positive |  |
| SLC31A1 | | AC090152.1 | | -0.474 | | | < 0.001 | negative |  |
| SLC31A1 | | AC092718.1 | | -0.433 | | | 0.001 | negative |  |
| SLC31A1 | | AC093227.1 | | -0.507 | | | < 0.001 | negative |  |
| SLC31A1 | | AC093278.2 | | 0.560 | | | < 0.001 | positive |  |
| SLC31A1 | | AC107204.1 | | -0.451 | | | < 0.001 | negative |  |
| SLC31A1 | | AC109361.1 | | -0.465 | | | < 0.001 | negative |  |
| SLC31A1 | | AC114810.1 | | -0.450 | | | < 0.001 | negative |  |
| SLC31A1 | | AC127024.2 | | -0.500 | | | < 0.001 | negative |  |
| SLC31A1 | | AC127024.3 | | -0.437 | | | 0.001 | negative |  |
| SLC31A1 | | AC138409.2 | | -0.465 | | | < 0.001 | negative |  |
| SLC31A1 | | AC211433.1 | | -0.479 | | | < 0.001 | negative |  |
| SLC31A1 | | AC211486.1 | | -0.436 | | | 0.001 | negative |  |
| SLC31A1 | | ADAMTSL4-AS1 | | 0.491 | | | < 0.001 | positive |  |
| SLC31A1 | | AF274858.1 | | -0.440 | | | 0.001 | negative |  |
| SLC31A1 | | AL031705.1 | | -0.478 | | | < 0.001 | negative |  |
| SLC31A1 | | AL133268.3 | | -0.455 | | | < 0.001 | negative |  |
| SLC31A1 | | AL137847.1 | | 0.463 | | | < 0.001 | positive |  |
| SLC31A1 | | AL162311.1 | | -0.428 | | | 0.001 | negative |  |
| SLC31A1 | | AL353743.1 | | -0.468 | | | < 0.001 | negative |  |
| SLC31A1 | | AL355353.2 | | -0.469 | | | < 0.001 | negative |  |
| SLC31A1 | | AL359232.1 | | -0.453 | | | < 0.001 | negative |  |
| SLC31A1 | | AL359715.1 | | -0.573 | | | < 0.001 | negative |  |
| SLC31A1 | | AL359715.3 | | -0.457 | | | < 0.001 | negative |  |
| SLC31A1 | | AL590652.1 | | -0.435 | | | 0.001 | negative |  |
| SLC31A1 | | AL591167.1 | | -0.460 | | | < 0.001 | negative |  |
| SLC31A1 | | AP003064.2 | | 0.529 | | | < 0.001 | positive |  |
| SLC31A1 | | B4GALT1-AS1 | | 0.744 | | | < 0.001 | positive |  |
| SLC31A1 | | CHROMR | | -0.552 | | | < 0.001 | negative |  |
| SLC31A1 | | DENND5B-AS1 | | -0.467 | | | < 0.001 | negative |  |
| SLC31A1 | | DNAJC3-DT | | -0.462 | | | < 0.001 | negative |  |
| SLC31A1 | | DPY19L2P3 | | -0.482 | | | < 0.001 | negative |  |
| SLC31A1 | | EMC3-AS1 | | -0.527 | | | < 0.001 | negative |  |
| SLC31A1 | | FOXN3-AS1 | | 0.493 | | | < 0.001 | positive |  |
| SLC31A1 | | GCC2-AS1 | | -0.558 | | | < 0.001 | negative |  |
| SLC31A1 | | GIHCG | | -0.447 | | | 0.001 | negative |  |
| SLC31A1 | | GUSBP1 | | -0.482 | | | < 0.001 | negative |  |
| SLC31A1 | | IPO5P1 | | -0.497 | | | < 0.001 | negative |  |
| SLC31A1 | | ITGB2-AS1 | | 0.662 | | | < 0.001 | positive |  |
| SLC31A1 | | KB-1674E1.2 | | -0.470 | | | < 0.001 | negative |  |
| SLC31A1 | | KCNQ5-AS1 | | -0.466 | | | < 0.001 | negative |  |
| SLC31A1 | | LINC00235 | | -0.480 | | | < 0.001 | negative |  |
| SLC31A1 | | LINC00539 | | -0.453 | | | < 0.001 | negative |  |
| SLC31A1 | | LINC00624 | | -0.513 | | | < 0.001 | negative |  |
| SLC31A1 | | LINC00630 | | -0.469 | | | < 0.001 | negative |  |
| SLC31A1 | | LINC00888 | | -0.494 | | | < 0.001 | negative |  |
| SLC31A1 | | LINC01353 | | 0.714 | | | < 0.001 | positive |  |
| SLC31A1 | | LINC01547 | | 0.574 | | | < 0.001 | positive |  |
| SLC31A1 | | LINC01624 | | 0.464 | | | < 0.001 | positive |  |
| SLC31A1 | | LINC01679 | | 0.534 | | | < 0.001 | positive |  |
| SLC31A1 | | LINC02175 | | -0.456 | | | < 0.001 | negative |  |
| SLC31A1 | | LINC02757 | | 0.743 | | | < 0.001 | positive |  |
| SLC31A1 | | LRRC37BP1 | | -0.436 | | | 0.001 | negative |  |
| SLC31A1 | | MIPEPP3 | | -0.513 | | | < 0.001 | negative |  |
| SLC31A1 | | MRPS30-DT | | -0.433 | | | 0.001 | negative |  |
| SLC31A1 | | NADK2-AS1 | | -0.443 | | | 0.001 | negative |  |
| SLC31A1 | | NAPSB | | 0.505 | | | < 0.001 | positive |  |
| SLC31A1 | | NNT-AS1 | | -0.485 | | | < 0.001 | negative |  |
| SLC31A1 | | PMS2P4 | | -0.537 | | | < 0.001 | negative |  |
| SLC31A1 | | PSMB8-AS1 | | 0.695 | | | < 0.001 | positive |  |
| SLC31A1 | | RP11-15H20.6 | | -0.499 | | | < 0.001 | negative |  |
| SLC31A1 | | RP11-327F22.1 | | 0.704 | | | < 0.001 | positive |  |
| SLC31A1 | | RP11-541P9.3 | | -0.475 | | | < 0.001 | negative |  |
| SLC31A1 | | RP11-58E21.3 | | 0.433 | | | 0.001 | positive |  |
| SLC31A1 | | RPS18P9 | | -0.549 | | | < 0.001 | negative |  |
| SLC31A1 | | RRN3P3 | | -0.471 | | | < 0.001 | negative |  |
| SLC31A1 | | SATB1-AS1 | | -0.573 | | | < 0.001 | negative |  |
| SLC31A1 | | SCOC-AS1 | | -0.497 | | | < 0.001 | negative |  |
| SLC31A1 | | SNHG29 | | -0.474 | | | < 0.001 | negative |  |
| SLC31A1 | | SPART-AS1 | | -0.464 | | | < 0.001 | negative |  |
| SLC31A1 | | SPDYE10P | | -0.431 | | | 0.001 | negative |  |
| SLC31A1 | | STAG3L1 | | -0.442 | | | 0.001 | negative |  |
| SLC31A1 | | STAG3L2 | | -0.583 | | | < 0.001 | negative |  |
| SLC31A1 | | STAG3L3 | | -0.580 | | | < 0.001 | negative |  |
| SLC31A1 | | STARD4-AS1 | | -0.466 | | | < 0.001 | negative |  |
| SLC31A1 | | SUZ12P1 | | -0.432 | | | 0.001 | negative |  |
| SLC31A1 | | SYNGAP1-AS1 | | -0.488 | | | < 0.001 | negative |  |
| SLC31A1 | | TRAF3IP2-AS1 | | -0.633 | | | < 0.001 | negative |  |
| SLC31A1 | | ZFAS1 | | -0.490 | | | < 0.001 | negative |  |
| SLC31A1 | | ZNF433-AS1 | | -0.596 | | | < 0.001 | negative |  |
| SLC31A1 | | ZNF561-AS1 | | -0.572 | | | < 0.001 | negative |  |
| SLC31A1 | | ZNF790-AS1 | | -0.615 | | | < 0.001 | negative |  |
| SLC31A1 | | ZNRF2P2 | | -0.476 | | | < 0.001 | negative |  |
| \| **Table S5. Multicollinearity test for the four cuproptosis-related lncRNAs** \| \| \| --- \| --- \| \| LncRNA \| VIF \| \| AC093278.2 \| 1.045514 \| \| AC133961.1 \| 1.024171 \| \| LINC01679 \| 1.42944 \| \| LINC02757 \| 1.502218 \|   VIF: Variance Inflation Factor   \| **Table S6. PH hypothesis for the four cuproptosis-related lncRNAs** \| \| \| \| --- \| --- \| --- \| \|  \| Chisq df \| *p* value \| \| AC093278.2 \| 1.257 1 \| 0.262 \| \| AC133961.1 \| 0.389 1 \| 0.533 \| \| LINC01679 \| 2.817 1 \| 0.093 \| \| LINC02757 \| 1.947 1 \| 0.163 \| \| GLOBAL \| 5.851 4 \| 0.211 \|  \| **Table S7. KEGG pathway analysis for AML patients with high- and low-risk analyzed by GSEA** \| \| \| \| \| --- \| --- \| --- \| --- \| \| NAME \| NES \| NOM  *P* value \| FDR \| \| **High-risk group** \|  \|  \|  \| \| KEGG_NOD_LIKE_RECEPTOR_SIGNALING_PATHWAY \| 2.173 \| 0.000 \| 0.025 \| \| KEGG_CHEMOKINE_SIGNALING_PATHWAY \| 2.164 \| 0.000 \| 0.013 \| \| KEGG_TOLL_LIKE_RECEPTOR_SIGNALING_PATHWAY \| 2.095 \| 0.000 \| 0.017 \| \| KEGG_CYTOKINE_CYTOKINE_RECEPTOR_INTERACTION \| 2.036 \| 0.000 \| 0.026 \| \| KEGG_FC_GAMMA_R_MEDIATED_PHAGOCYTOSIS \| 1.988 \| 0.002 \| 0.036 \| \| KEGG_NATURAL_KILLER_CELL_MEDIATED_CYTOTOXICITY \| 1.946 \| 0.002 \| 0.042 \| \| KEGG_B_CELL_RECEPTOR_SIGNALING_PATHWAY \| 1.922 \| 0.006 \| 0.042 \| \| KEGG_LEISHMANIA_INFECTION \| 1.894 \| 0.004 \| 0.047 \| \| KEGG_SYSTEMIC_LUPUS_ERYTHEMATOSUS \| 1.887 \| 0.010 \| 0.046 \| \| KEGG_LYSOSOME \| 1.880 \| 0.009 \| 0.044 \| \| KEGG_ANTIGEN_PROCESSING_AND_PRESENTATION \| 1.873 \| 0.008 \| 0.042 \| \| KEGG_HYPERTROPHIC_CARDIOMYOPATHY_HCM \| 1.853 \| 0.000 \| 0.048 \| \| KEGG_COMPLEMENT_AND_COAGULATION_CASCADES \| 1.849 \| 0.000 \| 0.046 \| \| KEGG_ENDOCYTOSIS \| 1.843 \| 0.002 \| 0.045 \| \| KEGG_EPITHELIAL_CELL_SIGNALING_IN_HELICOBACTER_PYLORI_INFECTION \| 1.839 \| 0.004 \| 0.044 \| \| KEGG_DILATED_CARDIOMYOPATHY \| 1.835 \| 0.000 \| 0.042 \| \| KEGG_T_CELL_RECEPTOR_SIGNALING_PATHWAY \| 1.831 \| 0.016 \| 0.041 \| \| KEGG_INTESTINAL_IMMUNE_NETWORK_FOR_IGA_PRODUCTION \| 1.818 \| 0.015 \| 0.046 \| \| KEGG_TYPE_I_DIABETES_MELLITUS \| 1.815 \| 0.004 \| 0.044 \| \| KEGG_ALLOGRAFT_REJECTION \| 1.803 \| 0.008 \| 0.047 \| \| KEGG_NEUROTROPHIN_SIGNALING_PATHWAY \| 1.796 \| 0.006 \| 0.048 \| \| KEGG_PATHOGENIC_ESCHERICHIA_COLI_INFECTION \| 1.788 \| 0.015 \| 0.049 \| \| KEGG_PANTOTHENATE_AND_COA_BIOSYNTHESIS \| 1.781 \| 0.006 \| 0.051 \| \| KEGG_CARDIAC_MUSCLE_CONTRACTION \| 1.773 \| 0.006 \| 0.052 \| \| KEGG_CYTOSOLIC_DNA_SENSING_PATHWAY \| 1.772 \| 0.006 \| 0.050 \| \| KEGG_GRAFT_VERSUS_HOST_DISEASE \| 1.768 \| 0.006 \| 0.050 \| \| KEGG_REGULATION_OF_ACTIN_CYTOSKELETON \| 1.767 \| 0.002 \| 0.048 \| \| KEGG_MAPK_SIGNALING_PATHWAY \| 1.756 \| 0.000 \| 0.050 \| \| KEGG_GNRH_SIGNALING_PATHWAY \| 1.741 \| 0.008 \| 0.054 \| \| KEGG_LEUKOCYTE_TRANSENDOTHELIAL_MIGRATION \| 1.738 \| 0.002 \| 0.053 \| \| KEGG_FC_EPSILON_RI_SIGNALING_PATHWAY \| 1.722 \| 0.004 \| 0.058 \| \| KEGG_VEGF_SIGNALING_PATHWAY \| 1.719 \| 0.008 \| 0.058 \| \| KEGG_SNARE_INTERACTIONS_IN_VESICULAR_TRANSPORT \| 1.713 \| 0.027 \| 0.059 \| \| KEGG_HEMATOPOIETIC_CELL_LINEAGE \| 1.713 \| 0.018 \| 0.057 \| \| KEGG_FRUCTOSE_AND_MANNOSE_METABOLISM \| 1.712 \| 0.012 \| 0.056 \| \| KEGG_JAK_STAT_SIGNALING_PATHWAY \| 1.706 \| 0.006 \| 0.057 \| \| KEGG_ARRHYTHMOGENIC_RIGHT_VENTRICULAR_CARDIOMYOPATHY_ARVC \| 1.706 \| 0.002 \| 0.055 \| \| KEGG_AMINO_SUGAR_AND_NUCLEOTIDE_SUGAR_METABOLISM \| 1.698 \| 0.028 \| 0.058 \| \| KEGG_PRION_DISEASES \| 1.679 \| 0.024 \| 0.065 \| \| KEGG_THYROID_CANCER \| 1.674 \| 0.016 \| 0.065 \| \| KEGG_APOPTOSIS \| 1.668 \| 0.030 \| 0.066 \| \| KEGG_AUTOIMMUNE_THYROID_DISEASE \| 1.666 \| 0.034 \| 0.065 \| \| KEGG_NOTCH_SIGNALING_PATHWAY \| 1.644 \| 0.033 \| 0.074 \| \| KEGG_VIBRIO_CHOLERAE_INFECTION \| 1.625 \| 0.024 \| 0.084 \| \| KEGG_CELL_ADHESION_MOLECULES_CAMS \| 1.623 \| 0.017 \| 0.083 \| \| KEGG_CALCIUM_SIGNALING_PATHWAY \| 1.618 \| 0.004 \| 0.083 \| \| KEGG_MELANOGENESIS \| 1.618 \| 0.015 \| 0.082 \| \| KEGG_SPHINGOLIPID_METABOLISM \| 1.613 \| 0.022 \| 0.082 \| \| KEGG_ECM_RECEPTOR_INTERACTION \| 1.606 \| 0.024 \| 0.085 \| \| KEGG_GLYCOSPHINGOLIPID_BIOSYNTHESIS_GANGLIO_SERIES \| 1.595 \| 0.017 \| 0.087 \| \| KEGG_FOCAL_ADHESION \| 1.594 \| 0.032 \| 0.085 \| \| KEGG_PPAR_SIGNALING_PATHWAY \| 1.591 \| 0.032 \| 0.086 \| \| KEGG_TYPE_II_DIABETES_MELLITUS \| 1.580 \| 0.018 \| 0.088 \| \| KEGG_WNT_SIGNALING_PATHWAY \| 1.579 \| 0.019 \| 0.087 \| \| KEGG_VASCULAR_SMOOTH_MUSCLE_CONTRACTION \| 1.560 \| 0.033 \| 0.096 \| \| KEGG_VASOPRESSIN_REGULATED_WATER_REABSORPTION \| 1.506 \| 0.048 \| 0.120 \| \| **Low-risk group** \|  \|  \|  \| \| KEGG_GLYCOSYLPHOSPHATIDYLINOSITOL_GPI_ANCHOR_BIOSYNTHESIS \| -1.828 \| 0.012 \| 0.231 \| \| KEGG_RNA_DEGRADATION \| -1.796 \| 0.026 \| 0.152 \| | | | | | | | | | |

| **Table S8. Drug sensitivity for AML patients with low- and high-risk** | | | | |
| --- | --- | --- | --- | --- |
| Drug name | Synonyms | Targets | Target Pathway | Lower IC50 |
| GNF-2 | KIN001-013 | BCR-ABL | ABL signaling | high risk group |
| CGP-082996 | CINK4, KIN001-021 | CDK4 | Cell cycle | high risk group |
| CGP-60474 | KIN001-019, CGP60474, CGP 60474 | CDK1,CDK2,CDK5,CDK7,CDK9, PKC | Cell cycle | high risk group |
| Seliciclib | Roscovitine, CYC-202, AL-39256 | CDK2, CDK7, CDK9 | Cell cycle | high risk group |
| Erlotinib | Tarceva, RG-1415, CP-358774, OSI-774, Ro-508231, R-1415 | EGFR | EGFR signaling | high risk group |
| BMS-536924 | BMS-536924 | IGF1R, IR | IGF1R signaling | high risk group |
| NSC-87877 | NSC 87877 | SHP-1 (PTPN6), SHP-2 (PTPN11) | Other | high risk group |
| A-770041 | KIN001-111 | LCK, FYN | Other, kinases | high risk group |
| Saracatinib | AZD0530, AZD-0530, AZ-10353926 | ABL, SRC | Other, kinases | high risk group |
| [GSK319347A](https://www.cancerrxgene.org/compound/GSK319347A/91) | KIN001-135, IKK-3 inhibitor | IKK | Other, kinases | high risk group |
| [WH-4-023](https://www.cancerrxgene.org/compound/WH-4-023/56) | KIN001-112 | SRC, LCK | Other, kinases | high risk group |
| WZ-1-84 | KIN001-123 | BMX | Other, kinases | high risk group |
| Rapamycin | AY-22989, Sirolimus, WY-090217, Torisel, Rapamune | MTORC1 | PI3K/MTOR signaling | high risk group |
| Bortezomib | PS-341, LDP-341, Velcade | Proteasome | Protein stability and degradation | high risk group |
| Elesclomol | STA-4783 | HSP90 | Protein stability and degradation | high risk group |
| Axitinib | AG-13736, Inlyta | PDGFR, KIT, VEGFR | RTK signaling | high risk group |
| Dasatinib | BMS-354825-03, BMS-354825, Sprycel | ABL, SRC, Ephrins, PDGFR, KIT | RTK signaling | high risk group |
| Lapatinib | Tykerb, Tyverb | EGFR, ERBB2 | RTK signaling | high risk group |
| NVP-TAE684 | NVP-TAE 684, TAE684, TAE-684 | ALK | RTK signaling | high risk group |
| Navitoclax | ABT-263, ABT263, ABT 263 | BCL2, BCL-XL, BCL-W | Apoptosis regulation | low risk group |
| [Obatoclax Mesylate](https://www.cancerrxgene.org/compound/Obatoclax%20Mesylate/182) | GX15-070MS, Obatoclax, GX15-070 | BCL2, BCL-XL, BCL-W, MCL1 | Apoptosis regulation | low risk group |
| AZD7762 | AZD-7762, AZD 7762 | CHEK1, CHEK2 | Cell cycle | low risk group |
| Palbociclib | PD0332991, PD-0332991, PF-00080665-73 | CDK4, CDK6 | Cell cycle | low risk group |
| Vorinostat | Zolinza, SAHA, suberanilohydroxamic acid, suberoylanilide hydroxamic acid, MK-0683 | HDAC inhibitor Class I, IIa, IIb, IV | Chromatin histone acetylation | low risk group |
| IPA-3 | IPA 3 | PAK1 | Cytoskeleton | low risk group |
| Camptothecin | Camptothecine, (+)-Camptothecin | TOP1 | DNA replication | low risk group |
| Etoposide | Etopophos, Vepesid, Eposin, VP-16 | TOP2 | DNA replication | low risk group |
| Gemcitabine | Gemzar, LY-188011 | Pyrimidine antimetabolite | DNA replication | low risk group |
| Methotrexate | Abitrexate, Amethopterin, Rheumatrex, Trexall, Folex | Antimetabolite | DNA replication | low risk group |
| Mitomycin-C | Mytozytrex, NSC-26980, MMC, Mitosol, Mitozytrex | DNA crosslinker | DNA replication | low risk group |
| Afatinib | BIBW2992, Tovok, Gilotrif | ERBB2, EGFR | EGFR signaling | low risk group |
| Gefitinib | ZD-1839, Iressa | EGFR | EGFR signaling | low risk group |
| Bicalutamide | ICI-176334, Casodex, Cosudex, ICI 176334 | AR | Hormone-related | low risk group |
| Linsitinib | OSI-906, ASP-7487 | IGF1R | IGF1R signaling | low risk group |
| VX-702 | VX702, VX 702 | p38 | JNK and p38 signaling | low risk group |
| Vinblastine | Velban | Microtubule destabiliser | Mitosis | low risk group |
| Tozasertib | MK 0457,MK-0457,MK-045, VX-680 VX 680 VX-68 | AURKA, AURKB, AURKC, others | Mitosis | low risk group |
| ZM447439 | ZM-447439, ZM 447439 | AURKA, AURKB | Mitosis | low risk group |
| Cytarabine | Ara-Cytidine, Arabinosyl Cytosine, U-19920 | Antimetabolite | Other | low risk group |
| Midostaurin | PKC412, benzoylstaurosporine, CGP-41251 | PKC, PPK, FLT1, c-FGR, others | Other | low risk group |
| QS11 | 944328-88-5 | ARFGAP1 | Other | low risk group |
| Tipifarnib | Zarnestra | Farnesyl-transferase (FNTA) | Other | low risk group |
| BAY-61-3606 | Syk Inhibitor, BAY-613606 | SYK | Other, kinases | low risk group |
| Bosutinib | SKI-606, Bosulif | SRC, ABL, TEC | Other, kinases | low risk group |
| Lestaurtinib | CEP-701, SP-924, SPM-924, A-154475, KT-555 | FLT3, JAK2, NTRK1, NTRK2, NTRK3 | Other, kinases | low risk group |
| GSK650394 | GSK-650394, GSK 650394 | SGK2, SGK3 | Other, kinases | low risk group |
| Nutlin-3a (-) | NA | MDM2 | p53 pathway | low risk group |
| AKT inhibitor VIII | Akti-1/2, KIN001-102 | AKT1, AKT2, AKT3 | PI3K/MTOR signaling | low risk group |
| AZD8055 | AZD-8055 | MTORC1, MTORC2 | PI3K/MTOR signaling | low risk group |
| MK-2206 | MK 2206, MK2206 | AKT1, AKT2 | PI3K/MTOR signaling | low risk group |
| Lenalidomide | CDC-501, CC-5013, Revlimid | CRBN | Protein stability and degradation | low risk group |
